# Trajectories, bifurcations, and pseudo-time in large clinical datasets: applications to myocardial infarction and diabetes data

**DOI:** 10.1093/gigascience/giaa128

**Published:** 2020-11-25

**Authors:** Sergey E Golovenkin, Jonathan Bac, Alexander Chervov, Evgeny M Mirkes, Yuliya V Orlova, Emmanuel Barillot, Alexander N Gorban, Andrei Zinovyev

**Affiliations:** Prof. V.F. Voino-Yasenetsky Krasnoyarsk State Medical University, 660022 Krasnoyarsk, Russia; Institut Curie, PSL Research University, F-75005 Paris, France; INSERM, U900, F-75005 Paris, France; CBIO-Centre for Computational Biology, Mines ParisTech, PSL Research University, 75006 Paris, France; Institut Curie, PSL Research University, F-75005 Paris, France; INSERM, U900, F-75005 Paris, France; CBIO-Centre for Computational Biology, Mines ParisTech, PSL Research University, 75006 Paris, France; Centre for Artificial Intelligence, Data Analytics and Modelling, University of Leicester, LE1 7RH Leicester, UK; Laboratory of advanced methods for high-dimensional data analysis, Lobachevsky University, 603000 Nizhny Novgorod, Russia; Prof. V.F. Voino-Yasenetsky Krasnoyarsk State Medical University, 660022 Krasnoyarsk, Russia; Institut Curie, PSL Research University, F-75005 Paris, France; INSERM, U900, F-75005 Paris, France; CBIO-Centre for Computational Biology, Mines ParisTech, PSL Research University, 75006 Paris, France; Centre for Artificial Intelligence, Data Analytics and Modelling, University of Leicester, LE1 7RH Leicester, UK; Laboratory of advanced methods for high-dimensional data analysis, Lobachevsky University, 603000 Nizhny Novgorod, Russia; Institut Curie, PSL Research University, F-75005 Paris, France; INSERM, U900, F-75005 Paris, France; CBIO-Centre for Computational Biology, Mines ParisTech, PSL Research University, 75006 Paris, France

**Keywords:** clinical data, clinical trajectory, patient disease pathway, dynamical diseases phenotyping, data analysis, principal trees, dimensionality reduction, pseudo-time, myocardial infarction, diabetes

## Abstract

**Background:**

Large observational clinical datasets are becoming increasingly available for mining associations between various disease traits and administered therapy. These datasets can be considered as representations of the landscape of all possible disease conditions, in which a concrete disease state develops through stereotypical routes, characterized by “points of no return" and “final states" (such as lethal or recovery states). Extracting this information directly from the data remains challenging, especially in the case of synchronic (with a short-term follow-up) observations.

**Results:**

Here we suggest a semi-supervised methodology for the analysis of large clinical datasets, characterized by mixed data types and missing values, through modeling the geometrical data structure as a bouquet of bifurcating clinical trajectories. The methodology is based on application of elastic principal graphs, which can address simultaneously the tasks of dimensionality reduction, data visualization, clustering, feature selection, and quantifying the geodesic distances (pseudo-time) in partially ordered sequences of observations. The methodology allows a patient to be positioned on a particular clinical trajectory (pathological scenario) and the degree of progression along it to be characterized with a qualitative estimate of the uncertainty of the prognosis. We developed a tool ClinTrajan for clinical trajectory analysis implemented in the Python programming language. We test the methodology in 2 large publicly available datasets: myocardial infarction complications and readmission of diabetic patients data.

**Conclusions:**

Our pseudo-time quantification-based approach makes it possible to apply the methods developed for dynamical disease phenotyping and illness trajectory analysis (diachronic data analysis) to synchronic observational data.

## Background

Large observational datasets are becoming increasingly available, reflecting the physiological state of observed individuals, their lifestyles, exposure to environmental factors, treatments received, and medical examinations undergone. From the big data point of view, each person’s life can be represented as a trajectory in a multidimensional space of qualitative or quantitative traits. Simultaneous analysis of a large number of such trajectories can reveal the most informative features whose dynamics is correlated with trajectory clusters, associations between various factors, and, potentially, the “points of no return," i.e., bifurcations representing important fate decisions.

The most important applications of such a framework are medical. The notion of “disease trajectory” as a person’s trajectory in the data space of various diagnoses (diseases quantified by their severity) accompanying the person’s life has emerged recently and became available for large-scale analyses in certain contexts [1,2]. For example, a dataset containing an electronic health registry collecting during 15 years and covering the whole population of Denmark, with 6.2 million individuals, has been analyzed with an objective of determining previously unreported disease comorbidities [1]. An ambitious “Data Health Hub" (https://www.health-data-hub.fr/) project has been recently launched in France with the aim of making available for machine learning–based analysis the collection of several decades-long population-wide anonymized health insurance records [3]. A formal review and meta-analysis of scientific texts using the concept of patient trajectory (or clinical pathway) based on disease management and care but also considering medico-economic aspects with a focus on myocardial infarction (MI) has been recently published [4].

Dynamical phenotyping is the conceptual paradigm underlying such studies, which can be applied at organismal and cellular scales [5–7]. It states that distinguishing various dynamical types of progression of a disease or a cellular program is more informative than classifying biological system states at any fixed moment of time because the type of dynamics is more closely related to the underlying hidden mechanism. From the machine learning point of view, this dictates different choices of methods, with clustering more adapted to the synchronic (snapshot) data [8] while more specific methods for trajectory analysis are needed in the case of diachronic (having important temporal aspect) data [9–13]. The dynamical phenotyping paradigm and accompanying data mining methodologies become even more important with wider introduction of various types of continuous health monitoring devices and apps [14].

However, examples of massive comprehensive and lifelong longitudinal clinical data are still rare. Most of the existing clinical datasets correspond to relatively short periods of patients’ stays inside hospitals, or during their treatment for a particular disease. In this sense, clinical datasets frequently represent a detailed but “static snapshot” rather than the dynamical picture of the individuals’ states. Nevertheless, one can hypothsize that such a snapshot, if sufficiently large, can sample the whole landscape of possible clinical states, with certain routes and branches corresponding to some average illness trajectories. Then each patient can be thought of as occupying a particular position along such a trajectory, where those patients following the same trajectory can be ranked according to their progression along it from the hypothetical least heavy state towards some extreme state.

This situation is reminiscent of some recent studies of molecular mechanisms of several highly dynamical biological processes such as development or differentiation, at the single-cell level. Indeed, profiling a snapshot of a cell population can capture individual cells in a variety of different states (e.g., map their progression through the cell cycle phases). This allows cellular trajectories to be reconstructed through sampling the dynamics of the underlying phenomenon without the need to follow each individual cell in time [15,16]. In this field, a plethora of machine learning–based methods have been recently suggested in order to capture the cellular trajectories and quantify progression along them in terms of “pseudo-time," representing the total number of molecular changes in the genome-wide profiles of individual cells [16] rather than physical time. Many of these methods are able to detect branching trajectories, where the branches can represent important bifurcations (cell fate decisions) in the dynamical molecular processes underlying differentiation of developmental programs.

The aim of the present study is to suggest and test a computational methodology for extracting clinical trajectories from sufficiently large synchronic clinical datasets. Clinical trajectory is a clinically relevant sequence of ordered patient phenotypes representing consecutive states of a developing disease and leading to some final state (i.e., a lethal outcome). Importantly, in our approach we do not assume that these are the same patient’s states, even if this can be so in the case when there exist some longitudinal observations. Each clinical trajectory can be characterized by its proper pseudo-time, which allows one to quantitatively characterize the degree of progression along the trajectory. Each clinical variable can be analyzed as a function of pseudo-time conditioned on a given clinical trajectory. We also assume that clinical trajectories can be characterized by branches (bifurcations), representing important decisive moments in the course of a disease.

Unlike the previously developed methodology of cell trajectory analysis in omics datasets, where the majority of the variables can be considered continuous and of similar nature (e.g., gene expression levels), the clinical datasets possess certain specifics that must be taken into account. Typical real-life clinical data are characterized by the following features: (i) they contain mixed data types (continuous, binary, ordinal, categorical variables, censored data); (ii) they typically contain missing values with non-uniform missingness pattern across the data matrix; and (iii) they do not have a uniquely defined labeling (part of the clinical variables can be used to define clinical groups, but this can be done in several meaningful ways). This means that an important integral part of the methodology should be procedures for quantifying and imputing missing values in mixed type datasets, making them amenable for further application of machine learning methods. The last feature (iii) suggests that unsupervised or semi-supervised methodology might play a more important and insightful role here than purely supervised methods.

We develop a methodology of clinical data analysis, based on modeling the multi-dimensional geometry of a clinical dataset as a “bouquet” of diverging clinical trajectories, starting from one or several quasi-normal (least severe) clinical states. As a concrete approach we exploit the methodology of elastic principal trees (EPT), which is a non-linear generalization of principal component analysis (PCA). Principal tree is a set of principal curves assembled in a tree-like structure, characterized by branching topology [17,18]. Principal trees can be constructed using the ElPiGraph computational tool, which has been previously exploited in determining branching trajectories in various genomics datasets (in particular, in single-cell omics data) [15,19,20]. As an unsupervised machine learning method, estimating elastic principal graphs solves several tasks simultaneously, namely, dimensionality reduction, data visualization, partitioning the data by the non-branching graph segments (analogous to clustering), and quantifying robust geodesic distances (pseudo-times) from one data point to another along the reconstructed principal graph. Unlike many other methods relying on heuristics for guessing the optimal graph topology (e.g., a tree) such as minimal spanning tree (MST), the elastic principal graph method optimizes the graph structure via application of topological grammars and gradient descent-like optimization in the discrete space of achievable graph structures (e.g., all possible tree-like graphs) [19].

The suggested method is implemented as a Python package, ClinTrajan, which can be easily used in the analysis of clinical datasets. We provide several reproducible Jupyter notebooks illustrating the different steps of the methodology. The figures in this article are directly copied from these notebooks. The methodology proved to be scalable to datasets containing hundreds of thousands of clinical observations, using an ordinary laptop, and can be scaled up further for even larger datasets.

## Data Description

In this study we apply the suggested methodology to 2 publicly available clinical datasets, 1 of moderate size (1,700 patients) and 1 of relatively large size (>100,000 patients).

### Complications of myocardial infarction database

MI is one of the most dangerous diseases. The wide spread of this disease over the past half century has made it one of the most acute problems of modern medicine. The incidence of MI remains high in all countries. This is especially true of the urban population of highly developed countries, exposed to chronic stress factors, unhealthful diet, and decreased physical activity. In the United States annually, >1 million people become ill with MI [21].

The course of the disease in patients with MI is diverse. MI can occur without complications or with complications that do not worsen the long-term prognosis. At the same time, roughly half of patients experience complications in the acute and subacute periods that lead to a worsening of the course of the disease and even death. Even an expert cannot always foresee the development of these complications. In this regard, predicting the complications of MI so that the necessary preventive measures can be carried out in a timely fashion could improve outcomes.

The database analyzed here was collected in the Krasnoyarsk Interdistrict Clinical Hospital (Russia) from 1992 through 1995 but has only recently been deposited to the public domain. The original database and its description can be downloaded [22]. It contains information about 1,700 patients characterized by 111 features describing the clinical phenotypes and 12 features representing possible complications of the MI disease (123 features in total). Previously, the dataset was a subject of machine learning method applications, including convolutional neural networks [23] and dimensionality reduction methods [24]. We believe that introducing this dataset, which exemplifies the specificity and difficulties of analyzing real-life clinical data, to the big data and machine learning research community should contribute to developing better treatment and subtyping strategies in cardiology and in clinical research in general [25].

A detailed description of the variable names with associated descriptive statistics is provided in the dataset description available online [22]. Here we provide Table 1 with the meaning of those variables that appear in the figures of the present article.

**Table 1.** Names of selected variables from the myocardial infarction complication dataset

Variable name	Meaning
General input values	
AGE	Age
DLIT_AG	Duration of arterial hypertension
ant_im	Presence of an anterior myocardial infarction (left ventricular)
FK_STENOK	Functional class of angina pectoris in the last year
GIPER_NA	Increase of sodium in serum (>150 mmol/L)
IBS_POST	Coronary heart disease in recent weeks, days before the admission time
inf_im	Presence of an inferior myocardial infarction (left ventricular)
lat_im	Presence of a lateral myocardial infarction (left ventricular)
K_BLOOD	Serum potassium content (mmol/L)
L_BLOOD	White blood cell count (billions per liter)
NA_BLOOD	Serum sodium content (mmol/L)
post_im	Presence of a posterior myocardial infarction
NA_R_1_n	Use of opioid drugs in the ICU in the first hours of the hospital period
NA_R_2_n	Use of opioid drugs in the ICU in the second day of the hospital period
NA_R_3_n	Use of opioid drugs in the ICU in the third day of the hospital period
NOT_NA_1_n	Use of NSAIDs in the ICU in the first hours of the hospital period
NOT_NA_2_n	Use of NSAIDs in the ICU in the second day of the hospital period
NOT_NA_3_n	Use of NSAIDs in the ICU in the third day of the hospital period
R_AB_1_n	Relapse of the pain in the first hours of the hospital period
R_AB_2_n	Relapse of the pain in the second day of the hospital period
R_AB_3_n	Relapse of the pain in the third day of the hospital period
TIME_B_S	Time elapsed from the beginning of the attack of CHD to the hospital
Inputs from anamnesis	
nr_03	Paroxysms of atrial fibrillation
nr_04	A persistent form of atrial fibrillation
nr_11	Observing of arrhythmia
np_10	Complete RBBB
STENOK_AN	Exertional angina pectoris
zab_leg_02	Obstructive chronic bronchitis
zab_leg_03	Bronchial asthma
zab_leg_06	Pulmonary tuberculosis
ZSN_A	Presence of chronic heart failure
Inputs for the time of admission to hospital	
n_p_ecg_p_06	Third-degree AV block on ECG
n_p_ecg_p_08	LBBB (posterior branch) on ECG
n_p_ecg_p_12	Complete RBBB on ECG
n_r_ecg_p_05	Paroxysms of atrial fibrillation on ECG
n_r_ecg_p_06	Persistent form of atrial fibrillation on ECG
n_r_ecg_p_08	Paroxysms of supraventricular tachycardia on ECG
ritm_ecg_p_01	Sinus ECG rhythm (HR between 60 and 90)
ritm_ecg_p_02	Atrial fibrillation in ECG rhythm
ritm_ecg_p_04	Atrial ECG rhythm
ritm_ecg_p_06	Idioventricular ECG rhythm
ritm_ecg_p_07	Sinus ECG rhythm (HR >90)
SVT_POST	Paroxysms of supraventricular tachycardia
Inputs for the time of admission to ICU	
D_AD_ORIT	Diastolic blood pressure (mm Hg)
S_AD_ORIT	Systolic blood pressure (mm Hg)
FIB_G_POST	Ventricular fibrillation
K_SH_POST	Cardiogenic shock
MP_TP_POST	Paroxysms of atrial fibrillation
O_L_POST	Pulmonary edema
Complications	
FIBR_PREDS	Atrial fibrillation
PREDS_TAH	Supraventricular tachycardia
JELUD_TAH	Ventricular tachycardia
FIBR_JELUD	Ventricular fibrillation
DRESSLER	Dressler syndrome
ZSN	Chronic heart failure
OTEK_LANC	Pulmonary edema
P_IM_STEN	Post-infarction angina
REC_IM	Relapse of the myocardial infarction
A_V_BLOK	Third-degree AV block
RAZRIV	Myocardial rupture
Cause of death	
LET_IS=0	Survive
LET_IS>0	Death with cause from 1 to 7
LET_IS_0	Survive
LET_IS_1	Cardiogenic shock
LET_IS_2	Pulmonary edema
LET_IS_3	Myocardial rupture
LET_IS_4	Progress of congestive heart failure
LET_IS_5	Thromboembolism
LET_IS_6	Asystole
LET_IS_7	Ventricular fibrillation

AV: atrioventricular; CHD: coronary heart disease; ECG: electrocardiogram; HR: heart rate; ICU: intensive care unit; LBBB: left bundle branch block; NSAID: nonsteroidal anti-inflammatory drug; RBBB: right bundle branch block.

### Diabetes readmission dataset

Together with MI, various diabetes-related clinical states such as hyperglycemia are widespread in the modern population. The management of hyperglycemia in hospitalized patients has a significant bearing on outcome, in terms of both morbidity and mortality [26]. An assembly and analysis of a large clinical database was undertaken to examine historical patterns of diabetes care in patients admitted to US hospitals and to inform future directions that might lead to improvements in patient safety [26]. In particular, the use of HbA1c as a marker of attention to diabetes care in a large number of individuals identified as having a diagnosis of diabetes mellitus was analyzed. A focus was on the readmission probability of a patient after leaving the hospital and its dependency on other clinical features that can be collected during hospitalization.

The dataset represents 10 years (1999–2008) of clinical care at 130 US hospitals and integrated delivery networks. It includes >50 features representing patient and hospital outcomes. The dataset can be downloaded from the UCI repository or from Kaggle (see Availability of Supporting Data and Materials). The data contain >100,000 hospitalization cases of patients with diabetes characterized by 55 attributes.

## Analyses

### ClinTrajan package for trajectory inference in large clinical datasets

We suggest the computational methodology of constructing principal trees in order to extract clinical trajectories from large-scale clinical datasets that take into account their specificity. The following steps of the analysis have been implemented:

- Univariate and multivariate quantification of nominal variables, including an original implementation of the optimal scaling procedure for ordinal values
- Several methods for imputation of missing values including 2 original implementations of singular value decomposition (SVD)-based imputers
- Constructing principal tree for a quantified clinical dataset
- Partitioning the data accordingly to the non-branching segments of the principal tree (analogue of clustering) and associating the segments with clinical variables
- Extracting clinical trajectories and associating the trajectories with clinical variables
- Visualization of clinical variables using principal trees and metro map data layouts [27]
- Pseudo-time plots of clinical variables along clinical trajectories, visualization of their bifurcations

### Myocardial infarction complications case study

#### Quantification of nominal values and imputation of missing data values

As a first step of pre-processing, 7 variables were removed from the initial MI complication data table as containing >30% of missing values. Next, 126 records were removed as containing >20% of the missing values. After this step, the data table contained 2.5% of missing values with 533 rows (34% of all clinical cases) having no missing values.

After the missing value filtering step, the data table of MI complications contained 84 binary, 9 continuous numerical, 22 ordinal, and 1 categorical variables. The large number of ordinal variables requires careful quantification (see Methods), which is not trivial given the large number of rows with missing values.

We considered the number of continuous numerical variables too small to apply the methodology of categorical principal component analysis (CatPCA) [28]. Therefore, for all ordinal and binary variables we first applied univariate quantification following the approach described in the Methods section. This quantification allowed the application of the “SVDComplete" imputation method for imputing the missing values, as described in the Methods. After all missing values were imputed, we could apply the optimal scaling approach for ordinal values, optimizing the pairwise correlations between them and between ordinal and continuous numerical variables. The 22 ordinal variables quantified in this way were further used for forming the data space. In addition, all variables were converted to *z*-scores.

#### Constructing elastic principal tree

The initial data space was formed by 123 variables. We evaluated the global intrinsic dimensions of the dataset using several methods implemented in Scikit-dimension Python package [29] and found that the majority of non-linear methods estimated the intrinsic dimensions in the range 10–15 while linear methods based on PCA gave much larger intrinsic dimension values (see Supplementary Figure S1). We compared the estimations of intrinsic dimensions with and without complication variables and found them to be similar, which indicates that there exists a certain level of dependency between the complication variables and the rest of the clinical variables. We also observed that the screen plot for this dataset is characterized by an elbow approximately at *n* = 12. As a result of this analysis, for further inference of the principal tree, we projected the dataset into the space of the first 12 principal components.

**Figure 1:**
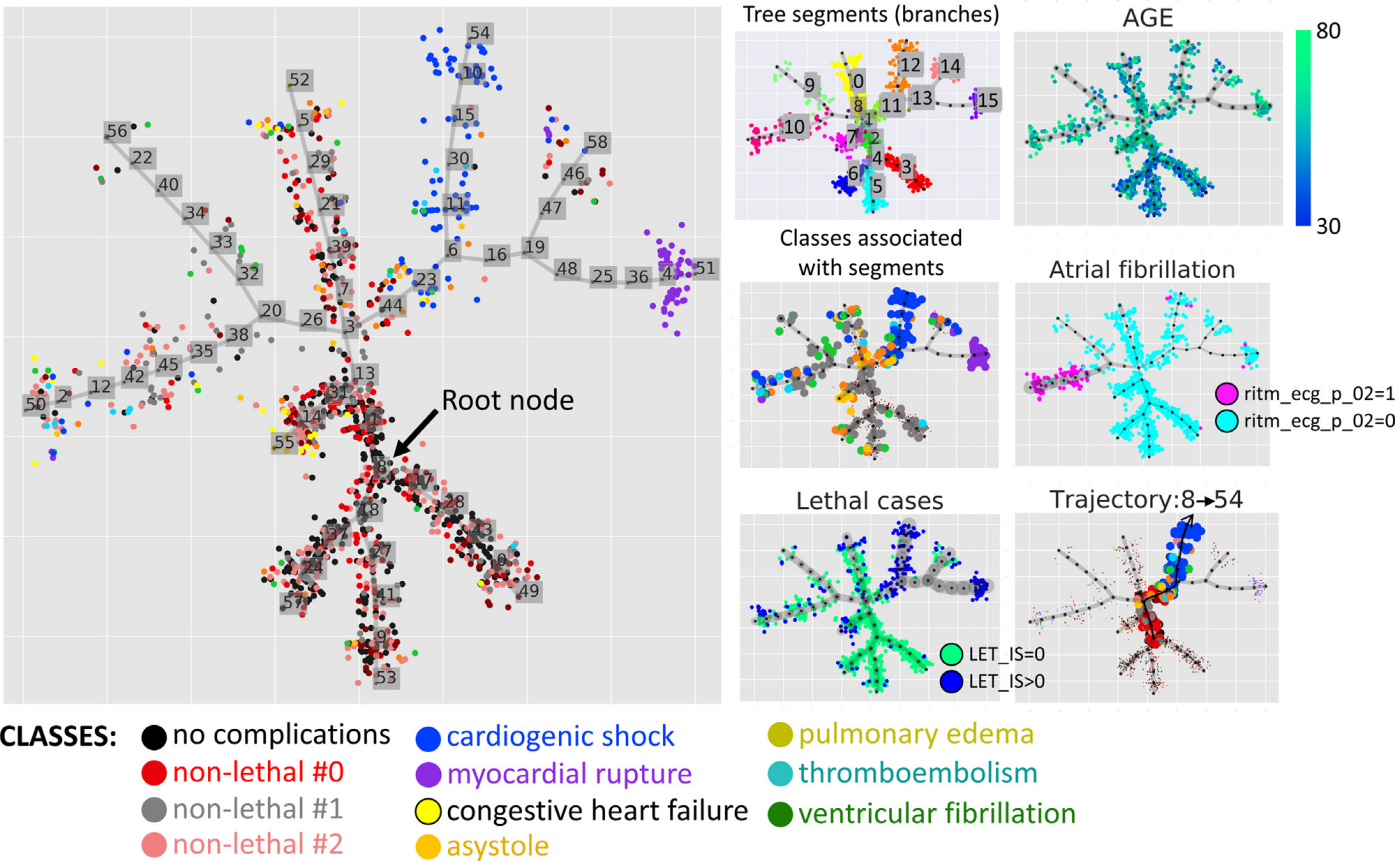
Principal tree recapitulating the multidimensional structure of the myocardial infarction complications dataset. Distribution of classes along the tree is visualized in the large panel. Various modes of data visualization are shown in the small panels. “Tree segments (branches)" shows partitioning (clustering) of data points accordingly to the linear fragments connecting branching points and/or leaf nodes (called “non-branching segments" in this work). “AGE" is an example of a continuous variable visualization using color gradient of data points. “Classes associated with segments" shows data points of only those classes that have statistical associations with ≥1 segments. “Atrial fibrillation" and “Lethal cases" show visualization of a binary variable, with edge width reflecting the trend (in the case of “Lethal cases" it can be interpreted as lethality risk estimate). “Trajectory 8→54" is a subset of data points, colored according to their classes and belonging to 1 particular clinical trajectory, having the node with the least risk of complications as the root node (Node 8) and the highest risk of cardiogenic shock as its final state (Node 54).

The elastic principal tree was computed using elastic principal graphs: ElPiGraph Python implementation as documented in the Jupyter notebook [30] and in the Section “Method of Elastic Principal Graphs (ElPiGraph)" of this article. The principal tree explained 52.4% of total variance, in contrast to the first 2 principal components, which explained 25.9%, and the first 5 principal components, which explained 54.0%. The obtained principal tree (shown in Fig. 1) was used to provide a 2D layout of the dataset, which can be used for visualization of various clinical variables and the results of analyses. Globally, the principal tree defined 3 terminal non-branching segments populated with non-lethal clinical cases [indicated as Nos. 3, 5, 6 in Fig. 1, panel “Tree segments (branches)"] and associated with younger patients (Fig. 1, panel “AGE"). Other terminal segments (Nos. 0, 7, 9, 10, 12, 14, 15) were characterized by various risks of lethality (Fig. 1, panel “Lethal cases"), with 2 terminal segments Nos. 12 and 15 being strongly enriched with lethal cases, caused by cardiogenic shock and myocardial rupture correspondingly.

Each node of the principal tree is connected with a subset of data points. We performed the enrichment analysis, based on application of the independence χ^2^ test, to determine the node that is the most strongly associated with the “no complication" class (black points in Fig. 1). The position of this node (No. 8) is indicated as “Root node" in Fig. 1, main panel.

#### Assigning data point classes

As mentioned above, the classes of the clinical observations can usually be defined by selecting a subset of clinical variables that represent some final readouts of a patient state. Thus, in the MI complications dataset, 12 clinical variables report the complications, with 11 of them representing binary variables and 1 the categorical variable LET_IS, whose value is 0 if there is no lethal outcome. Otherwise, LET_IS can take 1 of the 7 nominal values representing the cause of death. Following the methodology suggested in this study, the LET_IS variable is first made a subject of dummy coding, introducing 7 binary features. The resulting 18 binary variables were characterized by 158 unique combinations of 0/1 values, which appeared to be too many to define 1 class per each unique combination.

**Figure 2:**
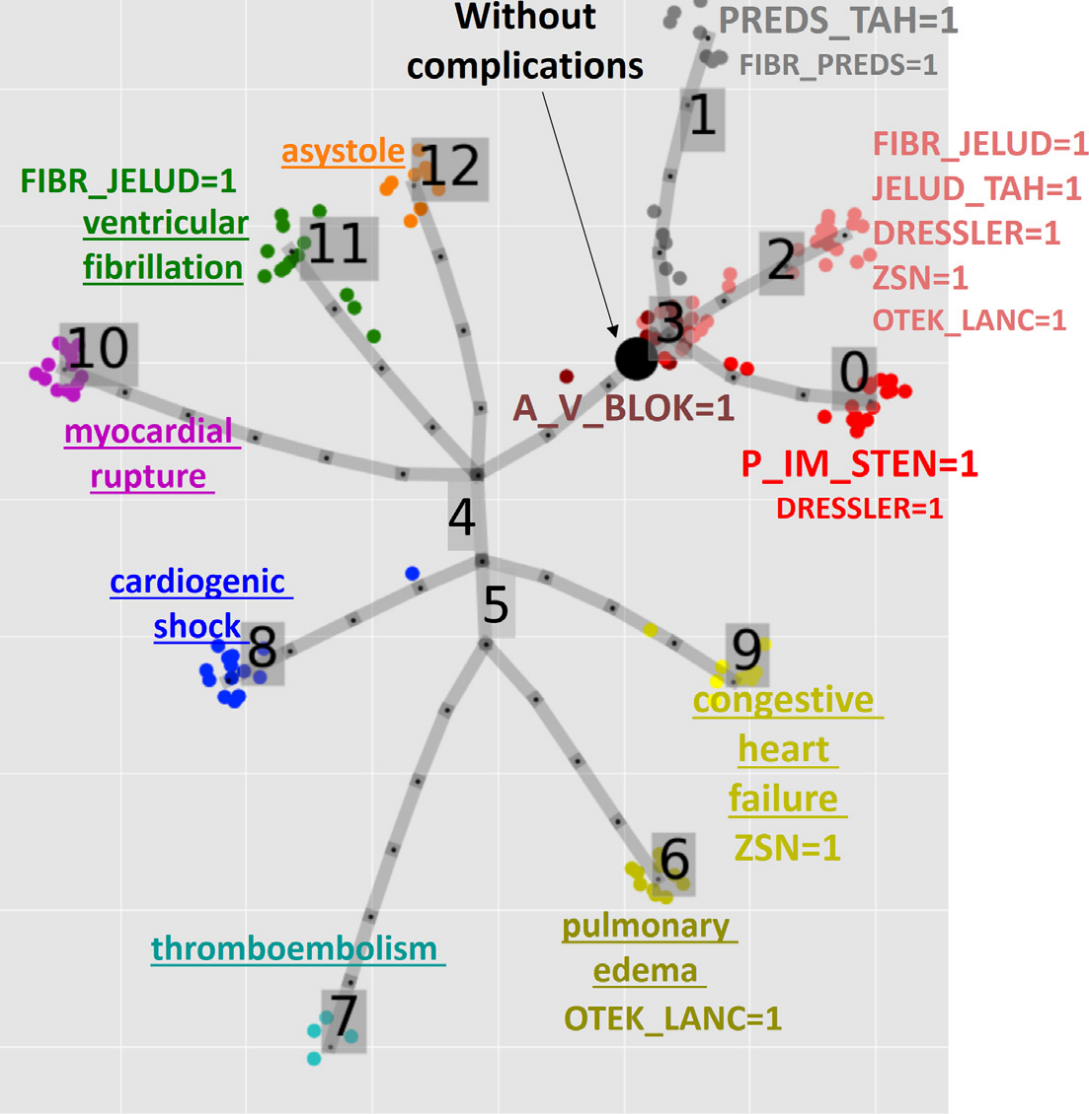
Defining classes of myocardial infarction complications using principal tree–based clustering. The labeling marks either the cause of death (underscored for lethal outcome classes) or a set of complication variables strongly overrepresented in the cluster, according to the χ^2^ test of independence; the size of the label reflects the significance of over-representation (the larger the label the more significant is the deviation from independence). A_V_BLOK: third-degree AV block; DRESSLER: Dressler syndrome; FIBR_JELUD: ventricular fibrillation; FIBR_PREDS: atrial fibrillation; JELUD_TAH: ventricular tachycardia; OTEK_LANC: pulmonary edema; P_IM_STEN: post-infarction angina; PREDS_TAH: supraventricular tachycardia; REC_IM: relapse of the myocardial infarction; ZSN: chronic heart failure.

Therefore, it was decided to reduce the number of distinct complication states to a more manageable number by clustering them. The table of 158 possible complications and 18 binary variables was analyzed by the method of elastic principal trees as described below and clustered into 11 clusters according to the principal tree non-branching segments (see Fig. 2). Seven of these clusters contained lethal outcomes and clearly corresponded to particular causes of death, which corresponds to non-zero values of the LET_IS variable. The non-lethal outcomes have been clustered into 4 classes (0, 1, 2, 3). Classes 1 and 2 appeared to be characterized by fibrillation and tachycardia but differed in the types (“1" corresponded to atrial fibrillation and tachycardia, while “2" had a tendency to be characterized by ventricular). Non-lethal Class 0 was distinguished by “P_IM_STEN" (post-infarction angina), and Class 3, by the presence of diagnosed “A_V_BLOCK" (third-degree atrioventricular block).

Besides this clustering, a particular non-lethal state was distinguished characterized by zero values of all complication variables. We distinguished this class as a separate “no complications class." In the complete dataset, it corresponded to 45% of clinical records (denoted as black points in Figs 1 and 2). In the rest of the analysis, all complication variables have been analyzed together with the clinical characteristics.

#### Dataset partitioning (clustering) by principal tree non-branching segments

The explicitly defined structure of the computed elastic principal tree allows the dataset to be partitioned in accordance with the projection of the data points on various internal and terminal segments as described in the “Methods" section and shown by color in Fig. 1, panel “Tree segments (branches)." Such partitioning can play a role of clustering with the advantage that the tree segments can recover non-spherical and non-linear data clusters. In addition, the data clusters, defined in such a way, are connected in a tree-like configuration, with junctions corresponding to the branching points, which can correspond to “points of no return" in the state of the patients.

**Figure 3:**
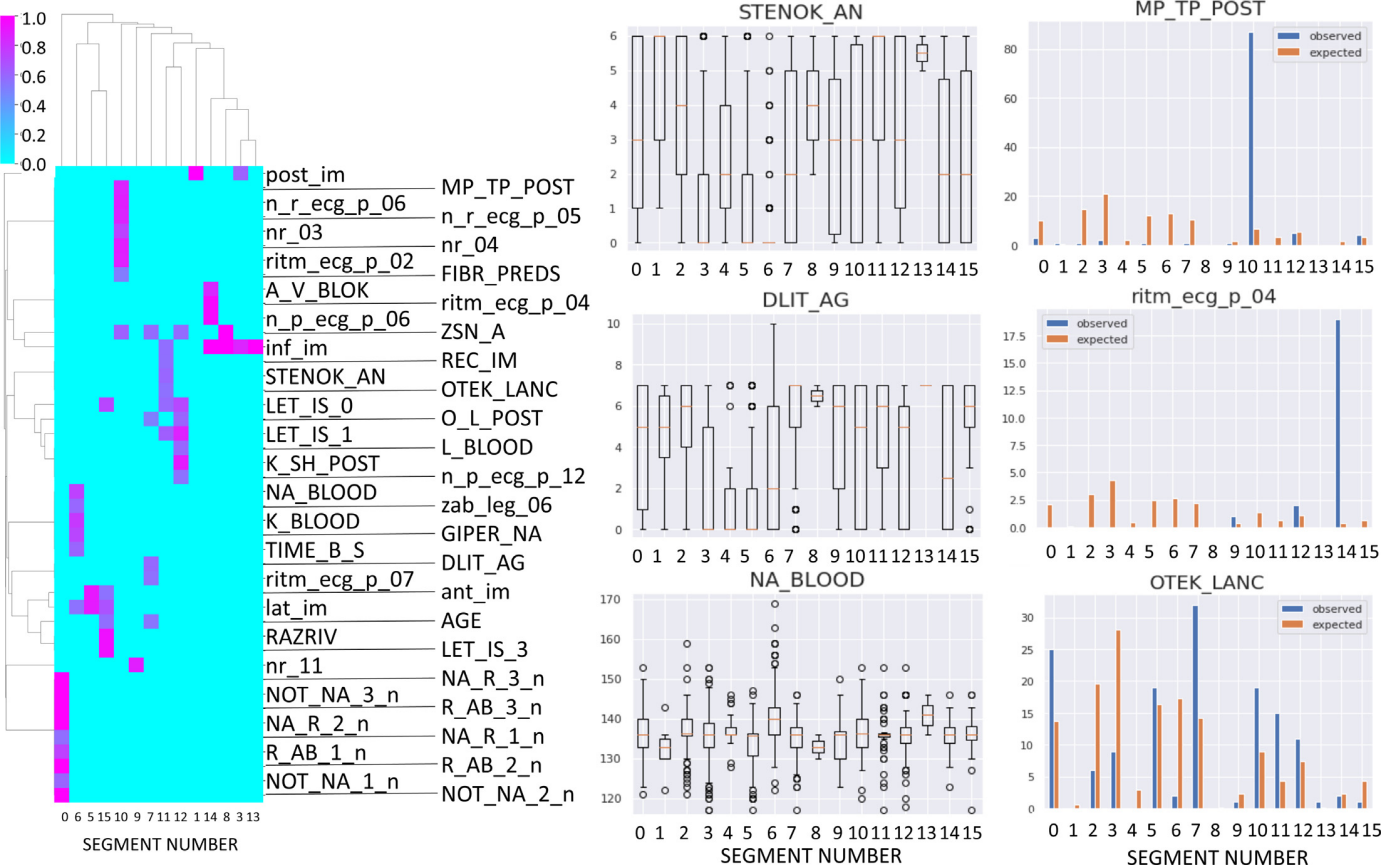
Association of principal tree segments (as shown in Fig. 1) with data variables. Right: Hierarchical clustering dendrogram of association scores. Right: 3 examples of strong associations with continuous/ordinal and binary variables. The following variables are shown: DLIT_AG: duration of arterial hypertension (years); MP_TP_POST: paroxysms of atrial fibrillation; NA_BLOOD: serum sodium content, mmol/L; OTEC_LANC, pulmonary edema; ritm_ecg_p_04, ECG rhythm at the time of admission to hospital, atrial; STENOK_AN, Exertional angina pectoris in the anamnesis. The meaning of other variable names is provided in the “Data description” section.

Each non-branching segment in the tree can be associated by enrichment analysis with either a data class or a variable. The points of the data classes that are associated with ≥1 tree segment are highlighted by size in Fig. 1, panel “Classes associated with segments." The results of enrichment analysis for all clinical variables are shown in Fig. 3. Briefly, we found that 44 clinical variables, including 8 complication variables, can be associated with ≥1 segment (Fig. 3) with reasonably high thresholds for either the deviation score (8) or ANOVA linear model coefficient (provided that the results of χ^2^ or ANOVA tests are statistically significant).

#### Analysis of clinical trajectories and pseudo-time

The non-branching segments of the principal tree are connected into trajectories, from the root node of the tree corresponding to the least frequency of complications to one of the leaf nodes representing some extreme states of the disease (some of which are connected with increased risk of lethality). Internal tree segments are shared between several trajectories, while the terminal segments correspond to a single trajectory. Consequently, each data point can be associated with one or more trajectories. The position of the data point on a trajectory is quantified by the value of pseudo-time characterizing the intrinsic geodesic distance from the root node, measured in the units of the number of tree edges. The value of pseudo-time is continuous because a data point can be projected on a tree edge, in between 2 nodes.

If a data point (clinical observation) is attributed to several trajectories, then it is characterized by the same pseudo-time value on each of them. This can be interpreted as the state of uncertainty from which several clinical scenarios can be developed in the further course of the disease, following 1 or several bifurcation points. Those clinical observations belonging to a single trajectory correspond to less uncertainty in the prognosis, with higher chances to end up in a terminal state.

**Figure 4:**
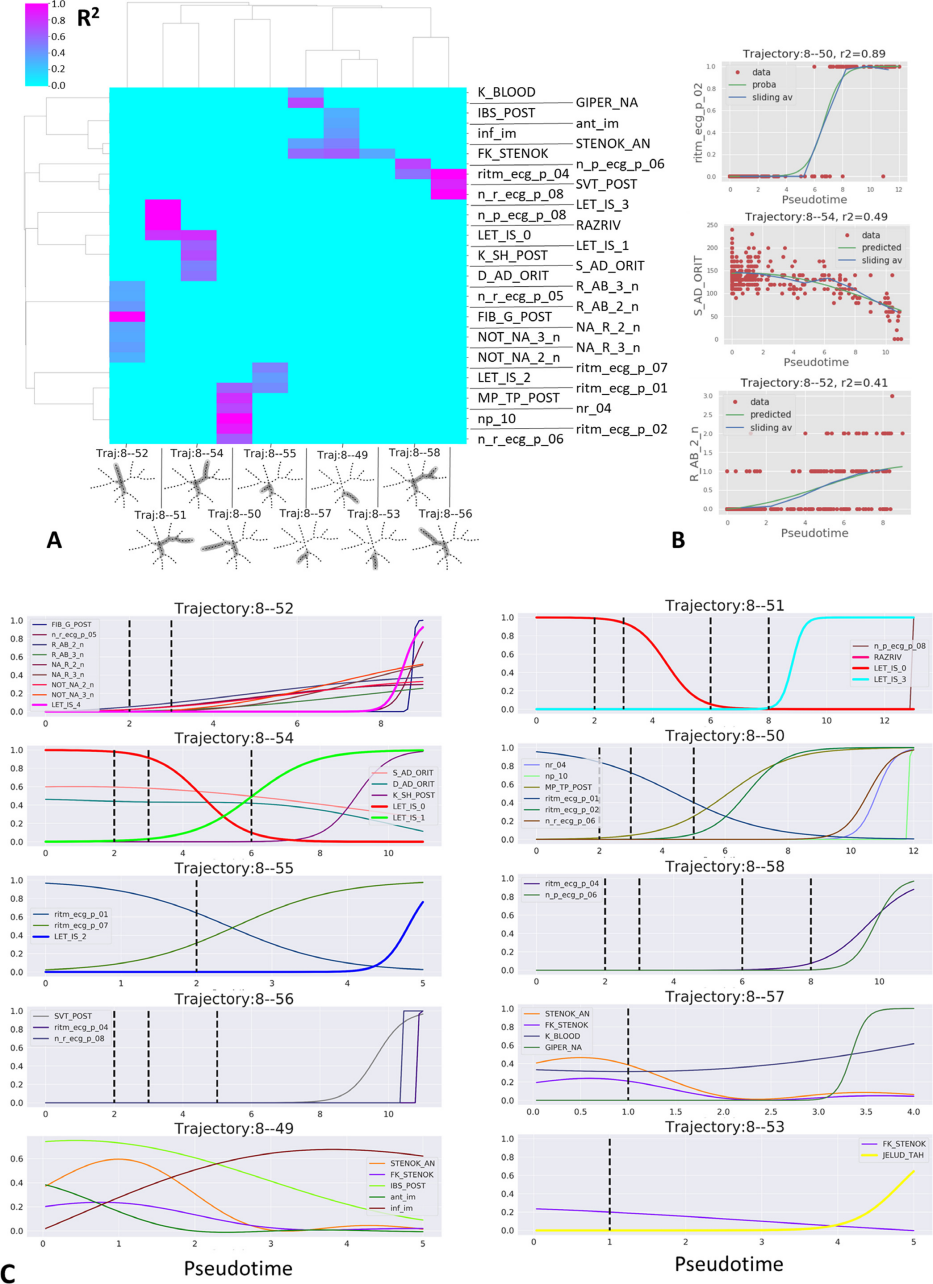
Clinical trajectory analysis of the myocardial infarction complications dataset. A, Visualization of *R*^2^ values for the regression between clinical variables and the pseudo-time along 10 clinical trajectories. B, Examples of regression analysis for binary (logistic regression), continuous, and ordinal (Gaussian kernel regression) clinical variables. C, Pseudo-time plots for clinical variables selected by regression analysis. For binary variables, the probability inferred by logistic regression is shown. For ordinal and continuous variables, non-linear regression line is shown. Complication variables associated with the clinical trajectories are shown with thick lines (e.g., LET_IS_0 represents the survival probability.) Vertical dashed lines indicate the positions of tree branching points along pseudo-time. The abscissa in the pseudo-time plots corresponds to the variable value scaled to unity for the total variable amplitude. The meaning of the variable names is provided in the “Data description” section.

To determine the factors affecting the choices between alternative clinical trajectories, it is necessary to associate clinical variables with each trajectory and determine the trend of their changes along them. Mathematically this corresponds to solving the regression problem connecting a clinical variable and the observation pseudo-time. Using this approach we identified 35 variables associated with pseudo-time with *R*^2^ > 0.3 for ≥1 trajectory (Fig. 4A and B). The pseudo-dynamics of these variables is shown in Fig. 4C. This analysis allows one to draw conclusions regarding the sequence of clinical variable changes leading to various complications. Thus, the 4 trajectories 8 → 52, 51, 54, 55 are associated with increasing risks of 4 distinct lethal outcomes (progress of congestive heart failure, myocardial rupture, cardiogenic shock, and pulmonary edema, respectively). Three trajectories (8 → 49, 53, 57) correspond to mild course of the disease associated with younger patients, with the risk of ventricular tachycardia increasing along the trajectory 8 → 53.

**Figure 5:**
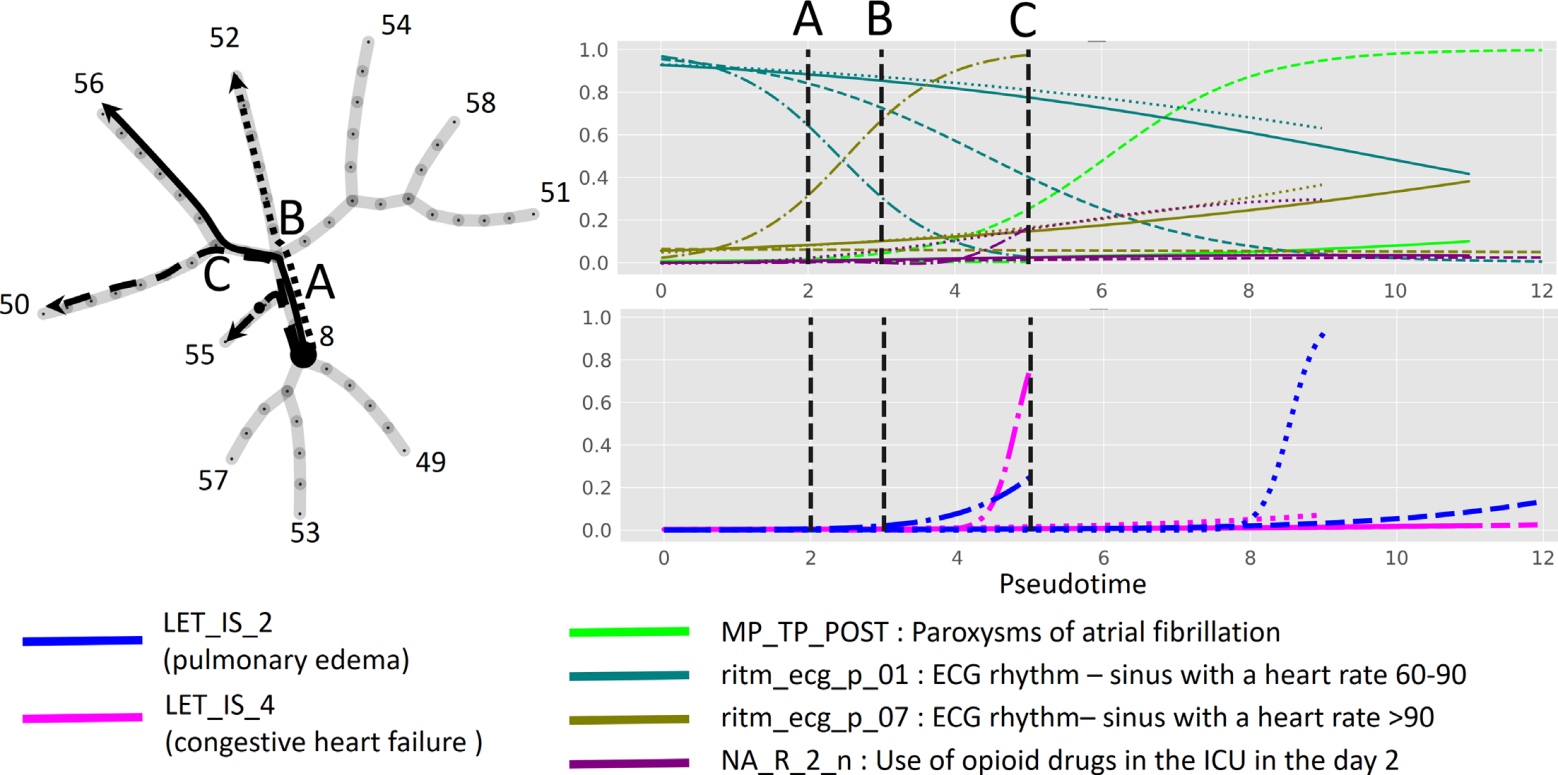
Example of bifurcating clinical trajectories. Four of 10 clinical trajectories are depicted for myocardial infarction complications data. The trajectories all share the internal segment 8-A and diverge at nodes A, B, C. Four selected binary clinical variables and 2 lethal outcome variables are shown as functions of 4 pseudo-time measurements, 1 per trajectory. The abscissa in the pseudo-time plots corresponds to the variable value scaled to unity for the total variable amplitude.

To illustrate the picture of decreasing uncertainty while the disease progresses along clinical trajectories, we focused on 4 trajectories 8 → 50, 52, 55, 56 sharing 1 or several internal tree segments. We selected several clinical variables and 2 lethal outcome variables associated with pseudo-time along these trajectories and showed them all in the same plot (Fig.   5). One can see that the pseudo-dynamics of some clinical variables estimated by logistic regression as having a probability of value “1" gradually diverge at the branching points of the principal tree.

The trajectory 8 → 55 is characterized by increasing sinus tachycardia after the bifurcation point A and increasing risk of congestive heart failure and to a lesser extent pulmonary edema. The trajectory 8 → 50 is characterized by the absence of sinus tachycardia with gradual decline in the variable “ECG rhythm - sinus with a heart rate 60–90" after the point B, and, after the bifurcation point C, rapid increase of the probability of paroxysms of atrial fibrillation. The prognosis along this trajectory is relatively favorable, as well as on the clinical trajectory 8 → 56, which is characterized by slow and incomplete decrease of the probability of “ECG rhythm - sinus with a heart rate 60–90" after the point C.

The trajectory 8 → 52 is characterized by high risk of pulmonary edema and gradual increase of sinus tachycardia. One of the distinguishing features of this trajectory is increased use of opioid and antiinflammatory drugs in the intensive care unit at days 2 and 3 after admission to the hospital, which is in turn connected to pain relapse (R_AB_2_n variable).

#### Predicting survival and lethal risk factors

Each clinical trajectory extracted from the analysis of synchronic clinical data is interpreted as a possible ordered sequence of states from the least severe condition to the extreme final point of the trajectory. Assuming that for a given patient state all the downstream points on the clinical trajectory represent possible future states of the patient, we can make a prediction of possible clinical risks connected with moving along this trajectory. In particular, this can be used for estimating lethal risks if such events are recorded in the clinical dataset. To evaluate the risks of a clinical event in the future, a well-developed methodology of survival analysis can be used, but using the pseudo-time value instead of the real time value. We call such analysis the pseudo-time survival analysis.

The pseudo-time quantified along different clinical trajectories might be incomparable in terms of the physical time. Therefore, the pseudo-time survival analysis should be performed for each trajectory individually, even if the estimated risks can be visualized together using the common pseudo-time axis.

**Figure 6:**
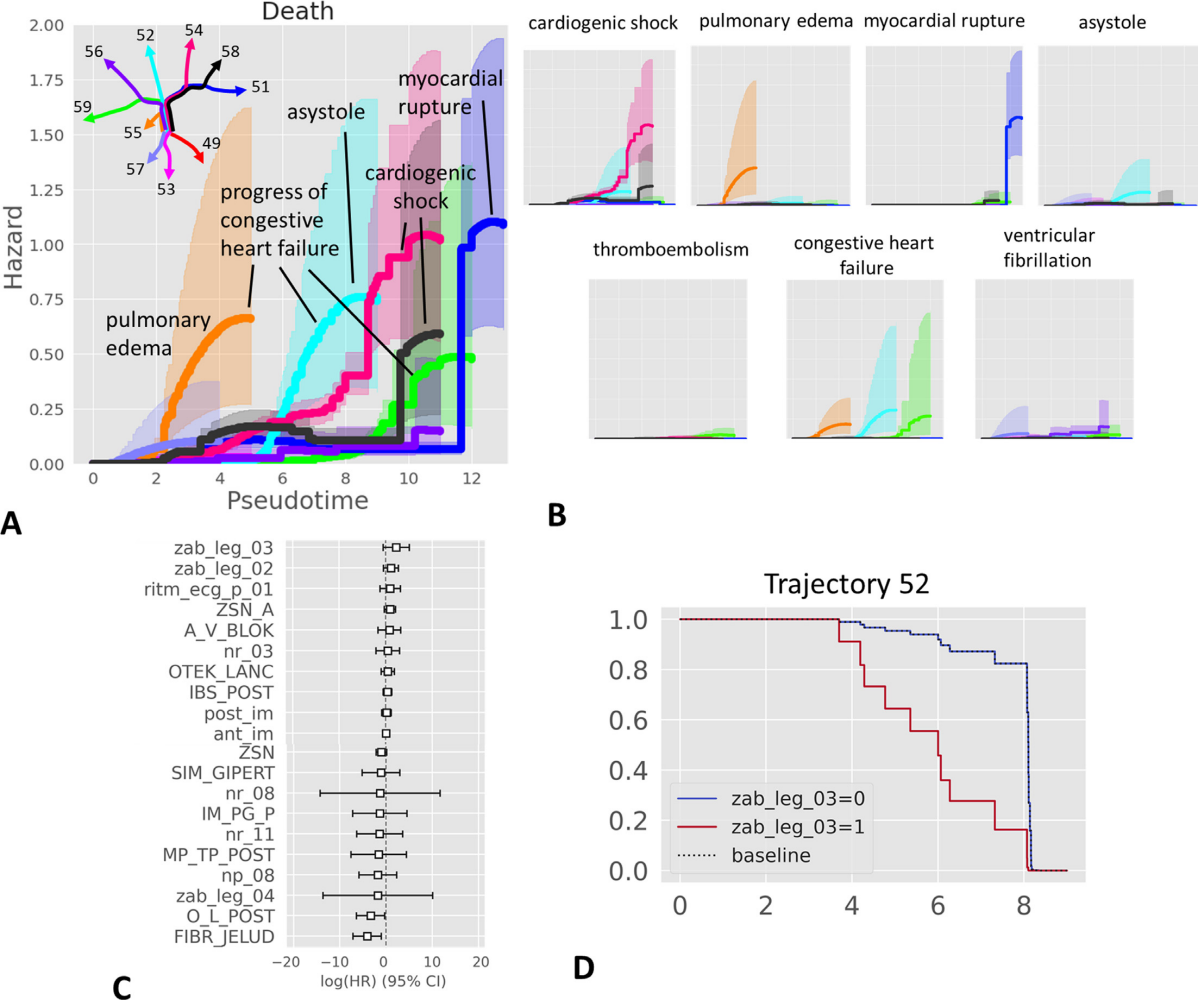
Pseudo-time survival analysis and determination of risk factors along clinical trajectories. A, Visualizing total hazards of death from myocardial infaction complications together with uncertainty of their estimate along different pseudo-time trajectories. The trajectories of the principal tree are denoted by different colors, corresponding to the color of the hazard plot. The dominant contribution of the cause of death to the total hazard is annotated by a label. B, Hazards of individual causes of death along various trajectories. The axis scale of each small plot here is identical to the plot shown in A. C, Example of survival regression for the data points along the trajectory ending with Node 52 (light blue in A). Only the 10 most significant positive and 10 most significant negative survival regression coefficients are shown. D, The effect of the top positive survival regression coefficient (zab_leg_03, meaning presence of bronchial asthma in the anamnesis) leads to different survival functions between 2 patient groups. Thus, presence of asthma in the anamnesis (zab_leg_03=1) worsens the survival along this particular trajectory associated with the risk of congestive heart failure and asystole (cardiac arrest). CI: confidence interval; HR: hazard ratio.

We applied a non-parametric estimator of the cumulative hazard rate function (see Methods) to quantify lethal risks along 10 identified clinical trajectories in the MI complication dataset (Fig. 6A). This analysis highlighted 6 of 10 trajectories as characterized by elevated hazard rates of lethality, which is a quantification of the distribution of lethal cases on the principal tree shown in Fig. 1. The total lethality risk can be decomposed into the risks resulting from a particular cause of death (1 of 7). Quantification of individual cause of death risks is shown in Fig. 6B. In this case, an event for the hazard function estimator is a particular cause of death. As a result, the increased risk of total lethality can be attributed to 1 or several particular causes of death (Fig. 6A).

Using the same assumptions, different risk factors affecting the risks along different clinical trajectories can be evaluated using the standard methodology of survival regression. As an example, we computed the survival regression for the set of patients along the trajectory ending in Node 52, associated with increased risks of congestive heart failure and asystole (cardiac arrest). The clinical variable making the largest positive contribution to the regression was the presence of bronchial asthma in the anamnesis, suggesting that it can be an aggravating factor along this particular clinical trajectory (Fig. 6C). Indeed, splitting this set of patients into 2 groups (with or without asthma in anamnesis) shows differential survival as a function of pseudo-time along this particular trajectory.

### Diabetes readmission case study

#### Clinical trajectories in large-scale observational diabetes data

To check whether the ClinTrajan package can be applied to larger datasets, we extracted clinical trajectories using a publicly available dataset, representing 10 years (1999–2008) of clinical care at 130 US hospitals and integrated delivery networks. The dataset contains 101,766 records satisfying the following conditions: (i) it is an inpatient encounter (a hospital admission); (ii) it is a diabetic encounter, i.e., one during which any kind of diabetes was entered to the system as a diagnosis; (iii) the length of stay was ≥1 day and ≤14 days; (iv) laboratory tests were performed during the encounter; and (v) medications were administered during the encounter. The data contain such attributes as patient race, sex, age, admission type, time in hospital, medical specialty of admitting physician, number of laboratory tests performed, HbA1c test result, diagnosis, number of medications, diabetic medications, and number of outpatient, inpatient, and emergency visits in the year before the hospitalization. In the supervised setting, the aim of the analysis of this dataset is usually to predict readmissions (“readmitted" variable) within 30 days after discharge from the hospital. In our analysis, we considered the readmitted variable as a part of the data space, in order to perform unsupervised analysis of the dataset with the aim of extracting clinical trajectories, some of them leading to the increased readmission likelihood.

The exact protocol for encoding the diabetes dataset is provided at the ClinTrajan github [30]. Importantly, we encoded several categorical variables as ordinal. In particular, the readmitted variable was encoded in 3 levels with 0 value corresponding to “No" (absence of recorded readmission), 1 to “>30 days," and 2 to “<30 days." The “A1Cresult" feature (related to the HbA1c test) was encoded in 2 variables. The first was binary, indicating absence (“None" value) or presence of the measured event. The second was the actual level of HbA1c: missing values corresponding to “None," and 3 levels encoding for the measured values, 0 for “Norm," 1 for “>7," and 2 for “>8." Because the A1Cresult field was not “None" in only 17% of patient records, this created a column containing 83% missing values, which were further imputed from the rest of the data. This was the only variable containing missing values. The age field was encoded as a 10-level ordinal variable according to 10 age intervals provided in the initial data table.

For encoding the 23 categorical fields of the dataset describing the administered medications and change in their dosage, we used the following schema. First, we kept only the 4 most frequently prescribed (in >10% of cases) medications: insulin (53% cases), metformin (20% cases), glipizide (12% cases), and glyburide (10% cases). Second, each medication field was encoded into 2 variables: a binary indicating the absence (“No" value) or presence (“Steady" or “Down" or “Up") of the treatment prescription, and a 3-level with 0 corresponding to either absence or no change in the treatment dose (“No" or “Steady"), −1 corresponding to decreased dose (“Down"), and +1 to increased dose (“Up").

We did not include some of the categorical variables such as admission type or diagnosis in the definition of the data space because they contained hundreds of different values, and we used them rather as annotations to be visualized on top of the constructed tree. We excluded 2.3% of records corresponding to the elapsed states (hence, without possibility of readmission) from the analysis, similarly to some previous analyses [31].

The resulting encoded dataset contained 22 variables (8 numerical, 7 ordinal, and 7 binary). We performed the data pre-processing similarly to the way it was done in the MI datasets, with imputing the missing values in the “A1Cresult_value" column and with further application of optimal scaling to ordinal values (see the corresponding Jupyter notebook [30]). The dimensionality of the dataset was reduced to 6 because it was the consensus value resulting from the application of several methods of intrinsic dimension estimation (see Supplementary Fig. 1), excluding outlying ID values.

**Figure 7:**
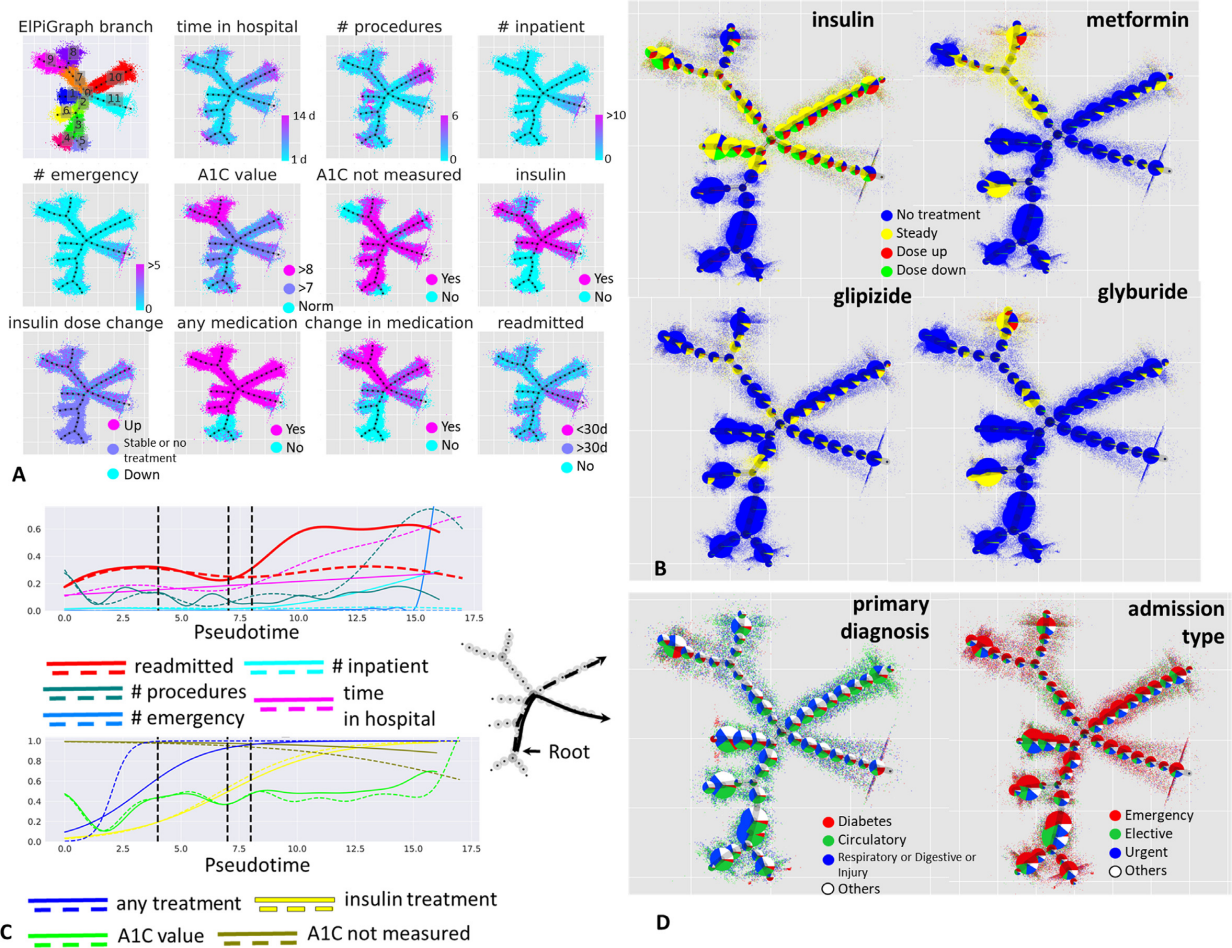
Analysis of clinical trajectories in large-scale diabetes dataset. A, Visualization of various clinical variables on top of the metro map layout of the principal tree. Partitioning the data according to the principal tree segments is also shown in the top left corner. B, Visualization of 4 categorical variables related to the administered drug treatments and their dose changes. Here, the data points are shown on a semi-transparent background, while on top of each graph node the relative proportions of the associated (the closest) data points are shown as a pie chart. The sizes of the pie charts are proportional to the number of points associated with each node. C, Pseudotemporal dynamics of clinical variables correlated to readmission- and long stay–associated clinical trajectories (shown as solid and dashed lines on the left). D, Visualization of the data table fields not participating in the construction of the principal tree, “primary diagnosis” on the left and “admission type” on the right.

The principal tree algorithm was applied with the same parameters as in the previous section. The construction of the principal tree with 50 nodes for the 6D dataset with 99,343 data points took ∼400 seconds on an ordinary laptop. The principal tree explained 64% of the total variance, in contrast to 47% for the first 2 principal components, with 4 principal components needed to explain the same percentage of variance as the principal tree. The tree contained 8 branching points (see Fig. 7A) with 1 fourth-order star. The principal tree–based data layout was used to visualize the values of data space variables (Fig. 7A) and some other variables from the annotation data (Fig. 7B and D), some of which did not participate in determining the structure of the principal tree.

As a root node in this case, we selected the middle node of one of the internal segments of the principal tree (segment No. 3 in Fig. 7A), which was characterized by the shortest times spent in the hospital, smallest number of all procedures, no history of inpatient stays or emergency calls in the preceding year, normal predicted (not measured) value of HbA1C, and absence of any medication. Therefore, this area of the principal tree was considered as corresponding to a quasi-normal state in terms of diabetes treatment.

Starting from this root node, the structure of the principal tree allowed us to define 8 distinct clinical trajectories. We focused on 2 of them, depicted in Fig. 7C as solid and dashed lines, together with the pseudo-time dependence of several selected clinical variables. One of these trajectories was the only one associated with the high readmission incidence, increasing with pseudo-time. It did not correspond, however, to the longest hospital stays, which was a feature of the second clinical trajectory considered. Therefore, we designate these clinical trajectories as “readmission-associated" and “long stay–associated." Unsurprisingly, the readmission-associated trajectory was characterized by an increasing number of inpatient and outpatient stays, as well as increasing number of emergency visits in the preceding year. This association must be interpreted by clinicians to attribute it either to objective clinical patient state requiring frequent return to the hospital or a psychologically motivated pattern of behavior. In favor of the objective cause, one can notice that the readmission-associated trajectory contains a different spectrum of primary diagnoses compared with the long stay–associated trajectory, where primary diagnoses related to the circulatory system dominate (Fig. 7D, left). We also note that elective hospitalizations were increasingly more frequent for the long stay–associated trajectory, while the pseudo-time of the readmission-associated trajectory correlates with increasing probability of admission by emergency (Fig. 7D, right).

The readmission-associated trajectory in this analysis can be considered an undesirable clinical scenario, the main source of burden on the medical system with respect to diabetes. By the trajectory-based analysis we confirmed previous conclusions [26] that the readmission-associated trajectory was connected with almost complete absence of HbA1C measurement (Fig. 7C), unlike the long stay–related trajectory, where up to 40% of patients underwent HbA1C testing at the final pseudo-time values. The predicted value of HbA1C along the readmission-related trajectory was “>7" (moderate elevation). Both readmission- and long stay–associated trajectories were characterized by receipt of insulin, with slightly more metformin indications along the long stay–associated trajectory. Importantly, the long stay–associated clinical trajectory is connected to the earlier, in terms of pseudo-time, “any treatment” variable dynamics (Fig. 7C, bottom panel).

#### Trajectory-based analysis of the relation between early readmission rate and the measured glycated hemoglobin HbA1c

In the original publication of the diabetes dataset, several observations were reported [26]. First, it was observed that the dependency of the early readmission (in <30 days) frequency estimate on the presence of an HbA1c measurement is conditional on the type of primary diagnosis (with 3 major types being diabetes and circulatory and respiratory diseases). Second, for the patients with diabetes as primary diagnosis, it was shown that not measuring the level of HbA1c is connected to increased risk of early readmission. Interestingly, from Fig. 1 of [26], one can conclude that, in patients with diabetes as primary diagnosis, high levels of measured HbA1c are connected to decreased readmission risk compared with the normal level of measured HbA1c. This paradoxical observation emerged from simple calculations of the early readmission frequency, as well as rate calculations adjusted for several clinical covariates.

**Figure 8:**
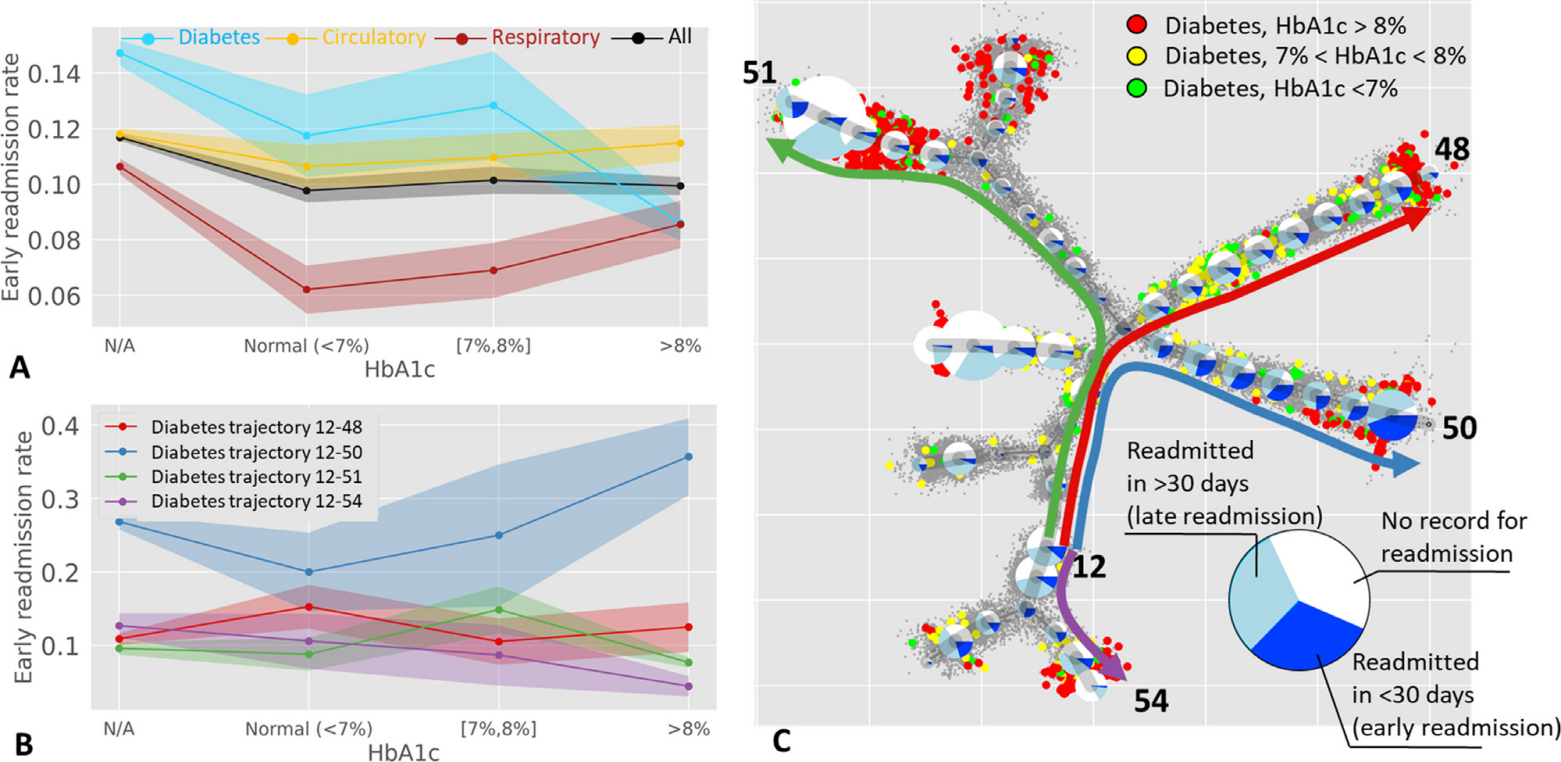
Relation between early readmission and measured glycated hemoglobin HbA1c. A, Global frequencies of early readmission (in <30 days) as a function of HbA1c measure. B, Frequencies of early readmission along 4 representative trajectories (shown in C), computed only for the patients with diabetes as primary diagnosis. Standard deviation interval is shown by shaded area for each curve in A and B. C, Pie charts show readmission frequencies along the clinical trajectories. The size of the pie charts reflects the number of patients with diabetes as primary diagnosis associated with each node of the principal tree. Bigger color data points show patients with diabetes as primary diagnosis, with known measured level of HbA1c (normal in green, medium in yellow, and high in red). Note that more severe cases of diabetes confirmed by HbA1c tend to be at the end of the clinical trajectories. The colors of the trajectories in C match those in the plots in B.

To illustrate the advantage of trajectory-based patient stratification, we recomputed the simple unadjusted estimations of the early readmission frequency as a function of measured HbA1c in sets of patients with different primary diagnosis (Fig. 8A). This reproduced the previously drawn conclusions from the original study [26]. The frequency of readmission appeared to be higher in the patients without measured HbA1c. Qualitatively similar to the previous publication, the readmission rate was significantly lower for the high values of HbA1c compared with normal levels (8.6% vs 11.8%). Note that the analyzed dataset has changed since its original publication, with >20,000 new patients being added. Noting this seeming paradox, we hypothesized that it can be explained by the heterogeneity of relationships between the levels of HbA1c and readmission, which can be captured in distinct clinical trajectories.

We looked at the cases of primary diagnosis of diabetes separately for each of the 8 clinical trajectories previously identified by applying the principal tree method (Fig. 8B). Strikingly, the dependence of early readmission on the measured levels of HbA1c is clearly different along different trajectories (Fig. 8C). For example, the trajectory ending with Node 50 (Fig. 8B and C, denoted as “Trajectory 12-50") was associated with higher risks of readmission. Absence of HbA1c measurement is still associated with a higher level of early readmission (26.8% compared with 20.0% for the cases with normal HbA1c level). However, along this trajectory the higher levels of measured HbA1c are associated with much higher levels of readmission (35.7%). There exists another trajectory (“Trajectory 12-54") where the dependence follows an opposite pattern (12.7% of early readmissions for unmeasured HbA1c vs 10.6% for normal levels of HbA1c and 4.4% for high levels of HbA1c).

Therefore, we can tentatively suggest that different trajectories in the diabetes data stratify the patients into clinically distinct scenarios, requiring different statistical models for anticipating the readmission rates. As a consequence, measuring HbA1c might have more clinical value in terms of estimating risks of early readmission along some trajectories and less along the others. For example, the patients with diabetes as primary diagnosis along Trajectory 12-50 are characterized by frequent readmissions, with more severe cases of diabetes leading to very frequent reamissions. Two other trajectories, exemplified in Fig. 8B and C, show much less of an effect of measuring HbA1c on readmission rates, and one of the trajectories shows an opposite trend, with more severe cases leading to less frequent readmission. As a consequence, we can suggest that measuring HbA1c is critical for determining the risk of early readmision for Trajectory 12-50 and Trajectory 12-54 but appears to be less important for Trajectory 12-48 and Trajectory 12-51.

Interpretation of distinct clinical scenarios must be performed by experts in the field of diabetes treatment. We can only hypothesize that the seeming decrease in the globally assessed readmission rates in patients with high measured HbA1c might be connected to the existence of a large subset of “stabilized" patients, with established supportive treatment. However, the stratification of patients into different clinical trajectories demonstrates that this is not a universal effect and that one can distinguish other patient clusters characterized by severe forms of diabetes characterized by relatively high rates of early readmission.

## Discussion

In this study we considered two rich and large publicly available observational clinical datasets from the most challenging areas of public health: cardiology and diabetes. Both datasets contain synchronic (related to a hospital stay) observations over a relatively large population of patients. Therefore, the traditional unsupervised machine learning approach for treating these data in order to classify clinical states is supposed to be some kind of clustering or manifold learning. We demonstrate that there exists an alternative approach that allowed us to represent these data as pseudo-diachronic, i.e., to reflect to some extent the temporal aspects. This opens a possibility to classify not only the states of particular patients but their hypothetical clinical trajectories arriving from the past and projected into the future. This in turn makes it possible to reason in terms of dynamical disease phenotyping, e.g., classifying clinical states in terms of the type of disease dynamics.

Identification of clinical trajectories is made possible by the use of the branching pseudo-time approach, consisting of modeling the geometry of the dataset as a “bouquet” of diverging trajectories, starting from 1 or several hypothetical quasi-normal (e.g., characterized by the least severe condition) disease states. The progression along a particular clinical trajectory can be quantified in terms of pseudo-time, reflecting the abstract accumulated amount of changes in the observed clinical traits. The main requirement for the possibility of such reconstruction is the existence of a sufficient number of observations (thousands) such that the individual variations in the clinical states would reveal the major non-linear routes along which they progress in real physical time.

Trajectory analysis from snapshot data is a widely used approach in modern molecular single-cell studies, where genome-wide measurements of individual cell states are inevitably destructive. Collecting information about a large number of cell states allows the underlying hidden cellular dynamics to be reconstructed without following each individual cell in physical time [15]. Dynamic phenotyping of cell states is a rapidly emerging concept in this scientific field [6]. ElPiGraph is an established general machine learning method that is widely used for the purpose of reconstructing cellular trajectories from single-cell data, in the form of principal trees or other more or less complex graph topologies [19]. Here we suggest applying ElPiGraph to the quantification of clinical trajectories in large clinical datasets, which requires adapting ElPiGraph to datasets characterized by mixed data types and the presence of non-randomly distributed missing values.

This effort resulted in the ClinTrajan Python package, which can be readily applied in the analysis of clinical datasets containing even millions of observations. In the real-life diabetes dataset considered here and containing >1 hundred thousands of observations, the analysis by ClinTrajan takes a few minutes on an ordinary laptop.

Use of ElPiGraph is the most relevant in the case when the hypothetical probability density function underlying the multi-dimensional data is characterized by certain archetypal features. Recall that classical phenotyping is, in its essence, cluster analysis of data. The application of standard clustering methods assumes thee existence of lumps and peaks in the density function: therefore, clustering looks for a set of principal points [8, 32].

Dynamical phenotyping has a different basic assumption that the point density is characterized by the existence of continuous 1D “ridges” that can diverge from or converge to each other in the data space. They can also connect local density peaks. In this case, the appropriate data approximation methods (such as ElPiGraph) look for principal curves and, more generally, branching principal trajectories, along which the data points are condensed [19,33]. The relevance of such a data model for dynamical phenotyping follows from the nature of a complex dynamical process, underlying disease progression, which develops in physical time and is sampled in the space of clinical characteristics.

Similar to cellular trajectories, the reconstructed clinical trajectories do not possess any natural orientation: therefore, orienting them necessitates expert-based decisions for choosing one or several root nodes in the principal tree. Also, the hypothetical dynamics of patients along the clinical trajectory does not have to be assumed to be irreversible. Some additional insights about orientation and reversibility can be obtained from a mix of synchronic and diachronic data, where individual patients can be represented not by simple data points but by more or less longitudinal observations represented by short trajectories. The best practices of using such data from the machine learning perspective remain to be established [10].

It appears interesting to relate the inferred pseudo-time to the physical time and use it to parametrize the obtained clinical trajectories. This raises for us an important challenge that can be approached in several ways.

One of them is related to the aging of patients. Indeed, chronological age represents the most basic way to rank the patients in a sequence that can potentially correlate to the clinical state (hence, define a clinical trajectory). However, the relationship between “biological" and “chronological" age remains complex, especially in the pathological context [34]. In our study we exploited chronological patient age as any other clinical variable, and observed that indeed age correlates to some clinical trajectories but not to others. Moreover, some clinical trajectories might be characterized by decreasing chronological age, which can be interpreted as an aggravating clinical picture specific to younger patients. We can imagine other ways of using the age variable: e.g., for learning the structure of the principal tree in a semi-supervised fashion. How to use chronological age in the most informative way when analyzing both longitudinal and synchronic data remains an open question [34].

A second approach to introducing physical time into the picture is using partially diachronic data as an additional annotation of a clinical dataset (the case of complete diachronic clinical data, representing longitudinal observations, is usually treated using a different and established set of approaches). One source of information that can be relatively easily obtained is identifying pairs of data points corresponding to 2 subsequent states of the same patient and recording the time lapse between the 2 states. For example, a fraction of the patients in a clinical dataset can be returning to the hospital, with a previous record included in the dataset, so this information must be available. If the number of such pairs is sufficiently large, then one can try to learn a monotonic function *F_k_* of pseudo-time along each trajectory *k*, predicting the actual temporal label for each observation. Note that the connection between pseudo-time and physical time can be different along different clinical trajectories. Moreover, the paired patient observation data can be used in the process of principal tree learning, by minimizing the number of paired patient observations belonging to distinct clinical trajectories.

Another limitation of the suggested approach is that the clinical trajectories are assumed to be diverging from some initial root state or states. In reality, convergence of clinical trajectories seems to be feasible (as in the case of the cellular trajectories). In this case, the model of the principal tree has to be generalized to some more general graph topologies (e.g., existence of few loops). In the case of the ElPiGraph method, such modifications are easy to introduce technically; however, introducing graph structures more complex than trees requires careful consideration to avoid creating data approximators whose complexity will be comparable to the complexity of the data themselves [35].

## Potential Implications

Quantification of clinical trajectories represents the first step in using the concept of dynamic clinical phenotyping for diagnostics and prognosis. Predicting the probabilities of future clinical states for a particular patient together with their uncertainties, using the knowledge of clinical trajectories, can be a natural next step for future studies. These approaches can consider clinical trajectories as a coarse-grained reconstruction of the state transition graph for a dynamic system, described by, e.g., continuous Markov chain equations. Some methodological ideas can be borrowed from recent omics data studies [36].

Recapitulating the multi-dimensional geometry of a clinical dataset in terms of clinical trajectories might open possibilities for efficient applications of other methods more oriented towards supervised machine learning. For example, it can potentially be used for learning the optimal treatment policy, based on the application of reinforcement learning as in [37].

The existing large clinical datasets are frequently collected as a result of multi-site studies. In the case of strong artifacts and biases caused by the application of significantly different practices for data collection or other factors, specific methods of correction should be applied, integrated into the data analysis or even in the study design [38]. However, dimensionality reduction methods based on averaging can in principle partially compensate for data heterogeneity if it can be modeled as a mixture of independent site effects that remain relatively small compared to the ranges of variable variations along the clinical trajectories. ElPiGraph in this respect has advantages over much more rigid PCA, being a nonlinear generalization of it for the case of datasets with complex geometries [19]. However, this aspect of ElPiGraph requires further specific investigation.

Overall, we believe that introducing trajectory-based methodology in the analysis of synchronic datasets might change the angle of view on their use for developing prognostic and diagnostic expert systems.

## Methods

### Implementation of the methodology

The ClinTrajan methodology is implemented in Python, packaged and openly available [30] together with Jupyter notebooks providing the exact protocols for applying the ClinTrajan package to several case studies. A detailed description of ClinTrajan functionality is provided on its website.

### Quantification of mixed-type datasets

Quantification of mixed-type datasets, i.e., assigning a numerical value for nominal variables, is a vast field in which many solutions have been suggested [39]. In the ClinTrajan package we used several popular ideas adapted to the aim of finding non-linear trajectories in the data.

First, for all non-binary categorical variables we suggest applying “dummy” encoding (or “one-hot” encoding), e.g., introducing new columns containing binary values 1 per category (one of the categories might be dropped as redundant). Alternatively, if there are enough numerical variables, CatPCA can be applied [28,40].

Second, for ordinal variables (including binary ones as a particular case) we suggest using either univariate or multivariate quantification. For univariate quantification we assume that the ordinal values are obtained by binning a “latent" numeric continuous variable possessing the standard normal distribution (zero mean and unit variance), following the approach described in [41]. Let us consider an ordinal variable *V*, which takes ordered values *v*_1_ < *v*_2_… < *v_m_*, and each *v_i_* value has *n_i_* counts in the dataset. We quantify *V* by the values

(1) \begin{eqnarray*}
x_i = \Phi ^{-1}\left( \sum \nolimits_{j=1}^{i-1}p_j+\frac{p_i}{2}\right), \end{eqnarray*}

where $p_i=n_i/N$, *N* is the total number of data points, and $\Phi (x) = 1/\sqrt{2\pi }\int _{-\infty }^xe^{-x^2/2}dx$.

If there exist many ordinal variables in the dataset, one can use multivariate methods to jointly quantify them. One of the most popular approaches is a particular variant of “optimal scaling," aiming at maximizing the sum of squared pairwise correlations between all variables, including numerical and ordinal ones [39]. The ClinTrajan package includes its own implementation of this variant of optimal scaling, which can be used to quantify ordinal variables in clinical datasets.

The advantage of multivariate ordinal variable quantification with respect to univariate is that it can decrease the intrinsic dimensionality of the resulting data point cloud, which can be beneficial for further application of manifold learning methods, including the method of ElPiGraph. The disadvantage of multivariate quantification of ordinal variables consists in the need to have a sufficiently large portion of data table rows without missing values. If this fraction is small, then it might be impossible to quantify certain ordinal levels because they will not be represented in this complete part of the dataset, while this might still be possible with univariate quantification.

Thus, imputing missing values requires quantification of ordinal variables, and multivariate quantification of them requires imputation of missing values. Therefore, in practice we apply a hybrid approach consisting in application of univariate quantification with further imputation of missing values and further application of multivariate quantification using the optimal scaling approach.

Last, we suggest transforming all continuous numerical variables to standard *z*-scores (i.e., centering and scaling) to make them comparable.

### Imputing missing values in mixed-type datasets

Real-life clinical datasets are almost always only partially complete and contain missing values. Typically, these values are not distributed uniformly across the rows and columns of the data matrix but rather form some non-random patterns, which can even be constructively used for the tasks of clinical data analysis [42]. A typical pattern is the existence of a column (or a row) containing an abnormally large number of missing values. One can define 2 parameters δ_row_ and δ_column_ as the maximally tolerable fraction of missing values in any row or column of the data matrix. The problem of finding the largest submatrix satisfying these constraints is not completely trivial but can be approximated by some simple iterative approaches. In practice, the trivial suboptimal solution consists in eliminating columns whose fraction of missing values is >δ_column_ and then eliminating the rows whose fraction of missing values is >δ_row_.

After constraining the maximum fraction of missing values in the data matrix, one can apply one of the available missing value imputation algorithms (imputers), which can also be classified into univariate and multivariate. For our purposes we advocate the use of multivariate imputers that allow us to avoid having strong data outliers destroying the manifold structure of the dataset. The standard Scikit-learn collection provides 2 types of imputers: nearest neighbors imputation and iterative multivariate imputation, which can in principle be used for this purpose. In the ClinTrajan package we add 2 alternative imputers based on the application of SVD of order *k*. The first one, which we designate “SVDComplete,” is applicable if the number of rows in the data matrix with no missing values is large enough (e.g., not much smaller than 50%). Then the standard SVD of order *k* is computed on the submatrix having only complete rows, and each data vector containing missing values is projected into the closest point of the hyperplane spanned by the first *k* principal components. The imputed value is then read out from the projected vector. For ordinal and binary variables, the imputed value can be additionally rounded to the closest discrete numerical value to avoid “fuzzy values” that do not correspond to any initial nominal value. The mutual exclusivity of binary variables encoding the categorical fields can also be taken into account. The second SVD-based imputer is called “SVDFull” and is based on computing SVD of order *k* for the full matrix with missing values, e.g., using the method suggested in [18,43]. After computing the principal vectors, the imputation is performed as in the SVDComplete imputer. The choice of *k* can be made either through applying cross-validation or by using a simple heuristics consisting in setting *k* to the value of the intrinsic dimensionality of the data. The intrinsic dimensionality can be estimated through the application of full-order SVD and analyzing the scree plot, or through a number of more sophisticated approaches [44].

### Method of Elastic Principal Graphs (ElPiGraph)

#### Computing the elastic principal graph

Elastic principal graphs are structured data approximators [17,18, 45, 46] consisting of nodes connected by edges. The graph nodes are embedded into the space of the data, minimizing the mean squared distance (MSD) to the data points, similarly to the *k*-means clustering algorithm. However, unlike unstructured *k*-means, the edges connecting the nodes are used to define the elastic energy term. This term is used to create penalties for edge stretching and bending of segments. To find the optimal graph structure, ElPiGraph uses a topological grammar (or, graph grammar) approach and gradient descent–based optimization of the graph topology, in the set of graph topologies that can be generated by a limited number of graph grammar operations.

An elastic principal graph is an undirected graph with a set of nodes *V* = {*V_i_*} and a set of edges *E* = {*E*^*i*^}. The set of nodes *V* is embedded in the multidimensional space. To denote the position of the node in the data space, we use the notation ϕ(*V_j_*), where ϕ(*V_j_*) is a map ϕ: *V* → *R*^*m*^. The optimization algorithm searches for such ϕ() that the sum of the data approximation term and the graph elastic energy is minimized. The optimization function is defined as follows:

(2) \begin{eqnarray*}
U^{\phi }(X,G) = \mathrm{MSD}^{\phi }(X,V)+U^{\phi }_E(G)+U^{\phi }_R(G), \end{eqnarray*}

where

(3) \begin{eqnarray*}
\mathrm{MSD}^{\phi }(X,V) = \frac{1}{|X|}\sum\nolimits_{i=1}^{|X|}\min \left[||X_i-\phi (V_{P(i)})||^2,R_0^2\right], \end{eqnarray*}

 

(4) \begin{eqnarray*}
U^{\phi }_E(G)=\sum\nolimits_{E^{i}}\lambda _{\mathrm{penalized}} \left(E^{i}\right) \left\{\phi \left[E^{i}(0)\right]-\phi \left[E^{i}(1)\right]\right\}^2, \end{eqnarray*}

 

(5) \begin{eqnarray*}
U^{\phi }_R(G)=\mu \sum \nolimits_{S^{j}} \left\{\phi [S^{j}(0)]-\frac{1}{\mathrm{deg}[S^{j}(0)]}\sum \nolimits_{i=1}^{\mathrm{deg}[S^{j}(0)]}\phi [S^{j}(i)] \right\}^2, \end{eqnarray*}

 

(6) \begin{eqnarray*}
\lambda _{\mathrm{penalized}}(E^{i}) = \lambda +\alpha \left[ \max \left(2,\mathrm{deg}[E^{i}(0)],\mathrm{deg}[E^{i}(1)]\right) -2\right], \end{eqnarray*}

where |*V*| is the number of elements in set *V, X* = {*X_i_*}, *i* = 1, …, |*X*| is the set of data points, *E*^*i*^(0) and *E*^*i*^(1) denote the 2 nodes of a graph edge *E*^*i*^, star *S*^*j*^ is a subgraph with central node *S*^*j*^(0) and several (>1) connected nodes (leaves), *S*^*j*^(0), …, *S*^*j*^(*k*) denote the nodes of a star *S*^*j*^ in the graph (where *S*^*j*^(0) is the central node to which all other nodes are connected), deg(*V_i_*) is a function returning the order *k* of the star with the central node *V_i_*, and *P*(*i*) = argmin_*j* = 1, …, |*V*|_‖*X_i_* − ϕ(*V_j_*)‖^2^ is a data point partitioning function associating each data point *X_i_* to the closest graph node *V*_*P*(*i*)_. *R*_0_, λ, μ, and α are parameters defined as follows: *R*_0_ is the trimming radius such that points farther than *R*_0_ from any node do not contribute to the optimization of the graph, λ is the edge-stretching elasticity modulo regularizing the total length of the graph edges and making their distribution close to equidistant in the multidimensional space, μ is the star-bending elasticity modulo controlling the deviation of the graph stars from harmonic configurations (for any star *S*^*j*^, if the embedding of its central node coincides with the mean of its leaves' embedding, the configuration is considered harmonic), and α is a coefficient of penalty for the topological complexity (existence of higher-order branchings) of the resulting graph.

Given a set of data points and a principal graph with nodes embedded into the original data space, a local minimum of *U*ϕ(*X, G*) can found by applying a splitting-type algorithm. Briefly, at each iteration given an initial guess of ϕ, the partitioning *P*(*i*) is computed, and then, given the *P*(*i*), *U*ϕ(*X, G*) is minimized by finding new node positions in the data space. A remarkable feature of ElPigraph is that the *U*ϕ(*X, G*) minimization problem is quadratic with respect to node coordinates and is reduced to solving a system of linear equations. Importantly, the convergence of this algorithm is proven [18,47].

Topological grammar rules define a set of possible transformations of the current graph topology. The graph configuration of this set possessing the minimal energy *U*ϕ(*X, G*) after fitting the candidate graph structures to the data is chosen as the locally best with a given number of nodes. Topological grammars are iteratively applied to the selected graph until given conditions are met [e.g., a fixed number of grammar applications or a given number of nodes is reached or the required approximation accuracy MSD^ϕ^(*X, V*) is achieved]. The graph learning process is reminiscent of a gradient descent–based optimization in the space of all possible graph structures achievable by applying a set of topological grammar rules (e.g., in the set of all possible trees).

One of the simplest graph grammars consists of the operations “add a node to node" and “bisect an edge," which generates a discrete space of tree-like graphs [19]. The resulting elastic principal graphs are called “elastic principal trees" in this case. In the ClinTranjan package we currently use only principal trees for quantifying trajectories and pseudo-time, even though using more general graph topologies is possible. The advantages of limiting the graph topology to trees are that it is easy to lay out the structure of the graph on a 2D plane and that any trajectory connecting 2 nodes of the graph is unique.

The resulting explicit tree structure can be studied independently of the data. Also, an arbitrary vector *x* (not necessarily belonging to the dataset *X*) can be projected onto the tree and receive a position in its intrinsic geodesic coordinates. The projection is achieved by finding the closest point on the principal graph as a piecewise linear manifold, composed of nodes and edges as linear segments connecting nodes. Therefore, the projection can end up in a node or on an edge. Furthermore, we define a projection function {*p*, ϵ} = Proj(*x, G*), returning a couple containing the index of the edge that is the closest to *x* and the position of the projection from the beginning of the edge *E*^*p*^(0) as a fraction of the edge length ϵ ∈ [0, 1]. Therefore, if ϵ = 0 then *x* is projected into *E*^*p*^(0) and if ϵ = 1 then the projection is in *E*^*p*^(1). If ϵ ∈ (0, 1) then the projection is on a linear segment, connecting *E*^*p*^(0) and *E*^*p*^(1).

A detailed description of ElPiGraph and related elastic principal graph approaches is available elsewhere [19]. The ElPiGraph package implemented in Python is available [48]. Implementations of ElPiGraph in R, Matlab, Java, and Scala are also available [49]. When analyzing the clinical datasets, the principal tree inference with ElPiGraph was performed using the following parameters: *R*_0_ = ∞, α = 0.01, μ = 0.1, λ = 0.05. After the initial principal tree was constructed, it was pruned and the terminal segments were extended. The pruning consists in eliminating the final terminal segments of the tree containing only 1 single edge. Extending the terminal segments consists in extrapolating the segment so that most of the data points will be projected on the edges of the terminal segment and not at its terminal node. Both functions are standard principal tree post-processing choices, implemented in the ElPiGraph package.

#### Partitioning (clustering) the data according to the principal graph segments

Embedding a graph to the data space allows us to partition (cluster) the dataset in several natural ways, e.g., by assigning each data point to the closest node or the closest edge. However, these ways do not fully suit our purposes because they do not reflect the intuition of “trajectory." So it is natural first to decompose the graph itself into linear fragments without branching (we call them non-branching graph segments or simply segments) and afterwards to cluster the dataset according to the closest segment. This is the idea of the data partitioning used in this article, and it is described in more detail below.

By the branching node in a graph we denote any node with connectivity degree >2, i.e., deg(*V_i_*) > 2, and by the leaf or terminal node of the graph we denote a node with degree <2, i.e., deg(*V_i_*) < 2.

**Figure 9:**
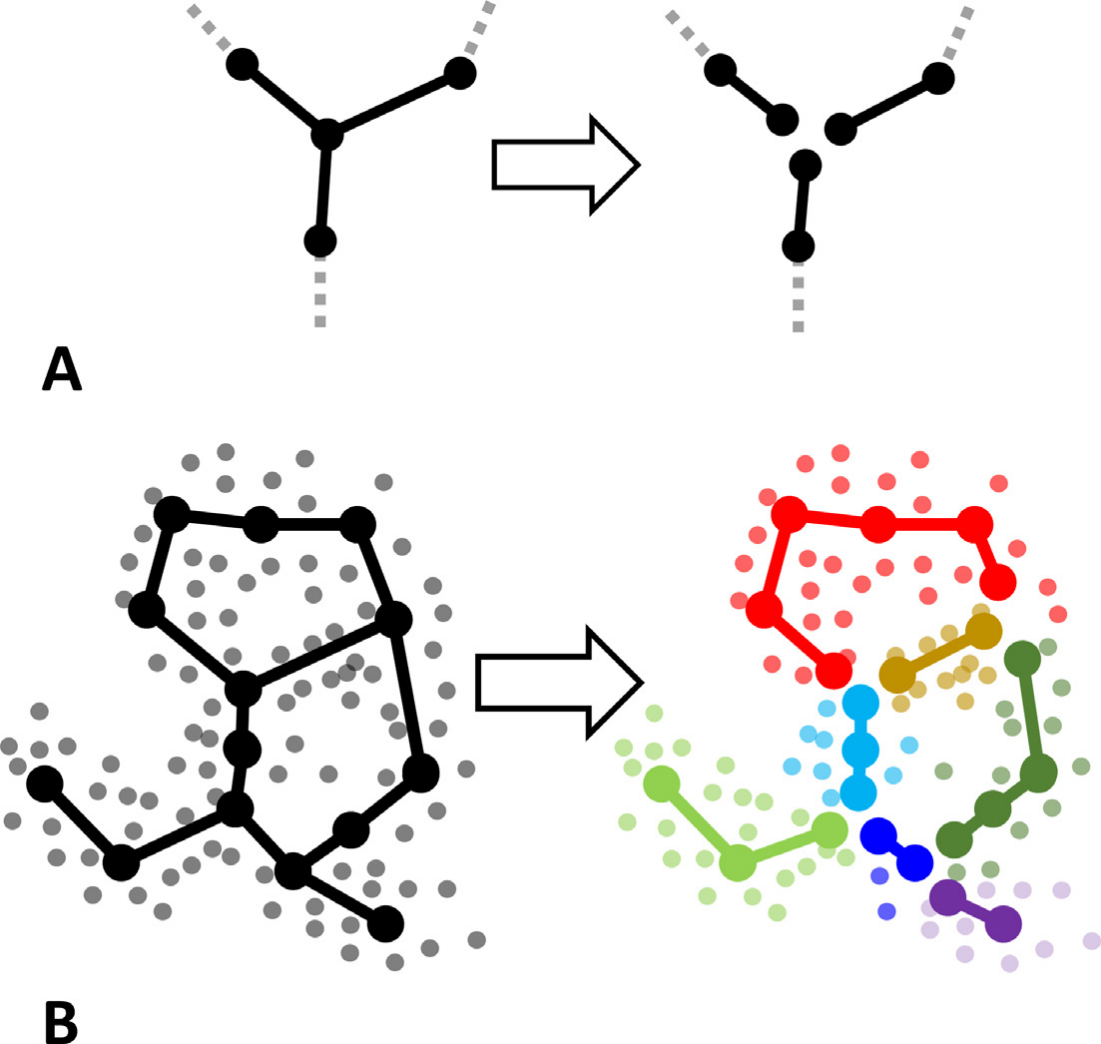
Decomposing a graph into non-branching segments and partitioning the data accordingly to the principal graph segments. A, The principal operation for segmenting the graph: each branching point of the graph (having degree >2) is multiplied and attached to the end of every edge composing the branching point such that the edges of the graph star are unglued and become disconnected. B, Toy example of a principal graph approximating a cloud of data points (shown in grey), and using its decomposition into non-branching segments for partitioning the data, which can be considered a kind of clustering (see text for details).

Let us call linear segment (or simply segment) of a graph such a path that connects a branching node to another branching or a leaf node and that does not contain any other branching nodes. Internal segments connect 2 branching nodes and the terminal segments connect a branching node to a leaf node (Fig.   9A). As one can see this definition reflects the intuition underlying the notion of the “segment”; we only need to specify several exceptional cases. For a graph that is an isolated cycle (not containing branching or leaf nodes), the whole cycle should be considered as a segment. The same is true if a graph contains several connected components that are cycles: then all of them are considered as separate segments. The other exceptional cases are nodes of degree zero (isolated nodes)—they also will be considered as separate segments. These exceptional cases cannot happen for connected principal trees, which are the main object of the present study; they are just mentioned for completeness.

Any graph can be uniquely split into “segments," which is not difficult to prove, especially for trees.

We coded in Python a version of the depth-first search algorithm to produce a split into segments for an arbitrary graph. The main difference from the classical depth-first search is a storage of visited edges (not only nodes) of the graph to correctly process possible cycles in the graph. The algorithm starts from any branching or leaf node and walks along edges in depth, joining them to the “current segment” until it meets a branching or a leaf node. Here, the current segment is terminated. In the case of a leaf node one returns from the recursion; the same goes for an already visited branching node. In the case of a new branching node (not visited before) one goes into a deeper level of the depth-first recursive process. After all edges of a graph are partitioned into segments, one can partition (or, cluster) the dataset according to the closest segment, which can be done in 2 ways. First, choose the edge nearest to a given data point and associate the point to the segment to which that edge belongs. However, a simpler approach is much more computationally efficient: calculate distances from data points to nodes and choose the segment that contains the nearest node. In case this node belongs to several segments (therefore it is a branching node), we choose the segment that contains the second-nearest node among all nodes that belong to the corresponding segments. If the number of nodes in the graph is large enough, then both approaches will produce (almost) identical results (Fig. 9B).

#### Dimensionality reduction and data visualization using principal graphs

To visualize the principal graph, each data point is first associated with the closest ElPiGraph edge in full dimensional space, and the distance to the projection onto the edge is recorded.

We then embed the graph structure in 2 dimensions by computing a force-directed layout with the Kamada-Kawai algorithm [50]. Each data point is placed orthogonally on a random side of its associated edge, at the distance proportional to the distance to the projection in the initial space. The proportionality constant is called the scattering parameter, which is adjusted by the user or can be optimized to best preserve the structure of the distances between the data points in the initial data space.

Edge widths can also be used to visualize the values of a variable or any function of the variables defined in the nodes of the graph.

#### Quantifying pseudo-time and extracting trajectories using principal trees

After computing the principal tree, a root node *V*_root_ has to be defined by the user, according to application-specific criteria. For example, it can correspond to the node of the graph closest to a set of data points enriched with those having the least disease severity.

The pseudo-time Pt(*x*) of an arbitrary vector *x* is defined as the total geodesic distance in the principal tree from *V*_root_ to the projection {*p*, ϵ} = Proj(*x, G*) of *x* on the graph. Algorithmically, we need to define which node of the edge *E*^*p*^ is the closest to *V*_root_ and add the ϵ accordingly, i.e.,

(7) \begin{eqnarray*}
\mathrm{Pt}(x) = \left\lbrace \begin{array}{ll}|V_{\mathrm{root}}\rightarrow E^p(0)|+\epsilon ,& \text{if } |V_{\mathrm{root}}\rightarrow E^p(0)|< |V_{\mathrm{root}}\rightarrow E^p(1)|\\ |V_{\mathrm{root}}\rightarrow E^p(0)|-\epsilon ,& \text{if } |V_{\mathrm{root}}\rightarrow E^p(0)|> |V_{\mathrm{root}}\rightarrow E^p(1)| \end{array} \right. \end{eqnarray*}

where |*V_i_* → *V_j_*| signifies the number of edges (length) of the trajectory *V_i_* → *V_j_*.

### Associating class labels and data variables and principal tree segments

Segment labeling of the data points induced by the structure of the principal graph represents a categorical label that can be associated to the dataset variables of various types.

To test whether there is an association of the tree segments to a categorical variable (including binary as a particular case), we used the standard independence χ^2^ test. If the test was significant, then we identified those segments that have the most unexpected value of the variable *k* by considering a simple deviation score:

(8) \begin{eqnarray*}
\mathrm{Deviation}_{k}(\text{value } j, \text{segment}\ i) = \frac{E^i_{kj} - O^i_{kj}}{E^i_{kj}}, \end{eqnarray*}

where $O^i_{kj}$ is the observed number of data points associated to the segment *i* having value *j* of the variable *k* and $E^i_{kj}$ is the expected number of occurrences of the value *j* of the variable *k*, from the standard independence assumption. Positive values of this score correspond to positive enrichment, and negative values, to negative enrichment.

To test for statistical association between tree segments and numerical variable (including ordinal as particular case), we used the standard ANOVA test representing the independent tree segment variable through the standard one-hot encoding into a set of binary variables. If the test was significant, then we evaluated the significance of each of the segments by looking at the value and the *P*-values of the generalized linear model coefficients for each segment. Positive values of the coefficients correspond to positive enrichment, and negative, to negative enrichment.

### Associating data variables and trajectories

For computing the score of association between a data variable *k* and a trajectory, we compute the *R*^2^ score of the regression:

(9) \begin{eqnarray*}
x^k = F(\mathrm{Pt}(x)), \text{for } x \in X_{V_{\mathrm{root}} \rightarrow V_j}, \end{eqnarray*}

where *V_j_* is one of the leaf nodes in the tree and Pt(*x*) is the pseudo-time value of the data point *x* computed from equation (7). For continuous variables, *F*() can be linear or a non-linear regression (e.g., the most popular Gaussian kernel regression). For binary variables, we fit *F*() by computing the logistic regression. We consider a variable *k* associated to the trajectory $X_{V_{\mathrm{root}} \rightarrow V_j}$ if *R*^2^ of the regression problem solution exceeds a certain threshold.

### Pseudo-time survival analysis

The survival analysis shown in Fig. 6 was performed using the Python package “lifelines" [51]. To estimate the hazard rate, we used the non-parametric Nelson-Aalen estimator of the cumulative hazard rate function implemented in the package. This estimator uses the formula [52]:

(10) \begin{eqnarray*}
H(t) = \sum \nolimits_{t_i\le t}\frac{d_i}{n_i}, \end{eqnarray*}

where *d_i_* is the number of observed events at time *t_i_* and *n_i_* is the total number of patients at risk at time *t_i_*. For each patient, instead of physical time *t_i_*, we used the value of pseudo-time computed along a particular trajectory.

For computing multivariate survival regression, we used the standard Cox model using the object “CoxPHFitter" from the same package.

## Availability of Source Code and Requirements

- Clinical trajectories (ClinTrajan)
- RRID:https://scicrunch.org/browse/resources/SCR_019018
- biotools: https://bio.tools/clintrajan
- Project home page: https://github.com/sysbio-curie/ClinTrajan
- Operating system(s): Platform independent
- Programming language: Python 3.*
- Other requirements: none
- License: LGPL

## Availability of Supporting Data and Materials

The myocardial infarction complication dataset can be downloaded from https://doi.org/10.25392/leicester.data.12045261.v3. The diabetes dataset can be downloaded from the UCI repository at https://archive.ics.uci.edu/ml/datasets/diabetes+130-us+hospitals+for+years+1999-2008 or from Kaggle at https://www.kaggle.com/brandao/diabetes. The dataset supporting this work is also openly available in the *GigaScience* repository, GigaDB [53].

## Additional Files

Supplementary Figure 1. Intrinsic dimensionality analysis of clinical datasets used in the study. The PCA-based estimation is defined here as the number of the eigenvalues of the covariance matrix exceeding λ_0_/*C*, where λ_0_ is the first (largest) eigenvalue and *C* is the maximal conditional number of the covariance matrix after dimensionality reduction (here *C* = 10). The computations were performed using the package Scikit-dimension Python package [29], where one can find the complete definitions of the methods and the corresponding references.

## Abbreviations

ANOVA: analysis of variance; CatPCA: categorical principal component analysis; ClinTrajan: Clinical Trajectory analysis; ElPiGraph: Elastic Principal Graph; MI: myocardial infarction; PCA: principal component analysis; SVD: singular value decomposition; UCI: University of California, Irvine.

## Competing Interests

The authors declare that they have no competing interests.

## Funding

This work has been partially supported by the Ministry of Science and Higher Education of the Russian Federation (project No. 14.Y26.31.0022), by the French government under management of Agence Nationale de la Recherche as part of the “Investissements d’Avenir” program, reference ANR-19-P3IA-0001 (PRAIRIE 3IA Institute), by the European Union’s Horizon 2020 program (grant No. 826121, iPC project), by the Association Science et Technologie, the Institut de Recherches Internationales Servier and the doctoral school Frontières de l’Innovation en Recherche et Education Programme Bettencourt.

## Authors' Contributions

A.Z., S.E.G., E.M.M., and A.N.G. designed the study. S.E.G., E.M.M., and Yu.V.O. prepared the myocardial infarction database, made it publicly available, and advised on its quantification. A.Z., J.B., A.Ch., E.M.M., A.N.G., and E.B. developed the methodology based on application of elastic principal graphs and A.Z., J.B., and A.Ch. implemented it in Python. J.B. packaged ClinTrajan. A.Z. and S.E.G. applied ClinTrajan to the clinical datasets. S.E.G., A.Z., A.N.G., and Yu.V.O. participated in the interpretation of the results. A.Z. and S.E.G. drafted the manuscript. All authors participated in editing and finalizing the text.

## Supplementary Material

giaa128_GIGA-D-20-00191_Original_Submission

giaa128_GIGA-D-20-00191_Revision_1

giaa128_GIGA-D-20-00191_Revision_2

giaa128_Response_to_Reviewer_Comments_Original_Submission

giaa128_Response_to_Reviewer_Comments_Revision_1

giaa128_Reviewer_1_Report_Original_Submission Chris Armit -- 7/15/2020 Reviewed

giaa128_Reviewer_1_Report_Revision_1 Chris Armit -- 10/13/2020 Reviewed

giaa128_Reviewer_2_Report_Original_Submission Zhengping Che -- 8/5/2020 Reviewed

giaa128_Reviewer_3_Report_Original_Submission Mark Schiebler, M.D. -- 8/28/2020 Reviewed

giaa128_Supplemental_File
