# Supplementary figures and images for: Trajectories, bifurcations, and pseudo-time in large clinical datasets: applications to myocardial infarction and diabetes data
Source: Gigascience. 2020 Nov 25;9(11):giaa128. doi: 10.1093/gigascience/giaa128 (PMC7688475; doi:10.1093/gigascience/giaa128)

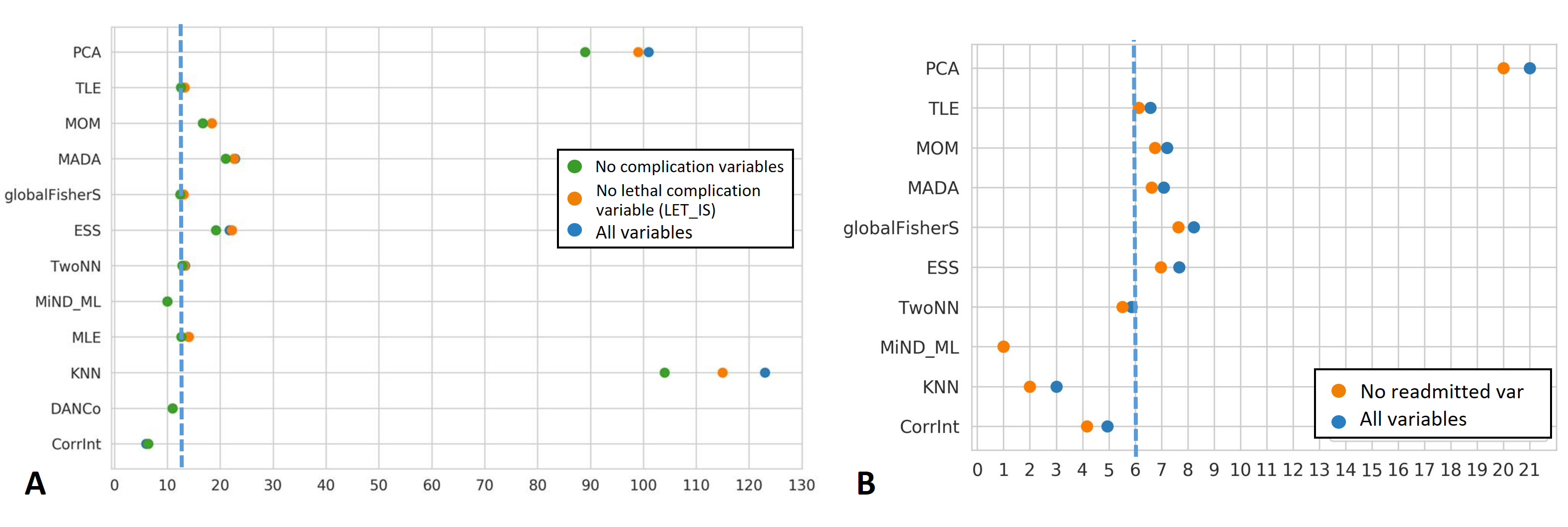

Supplement: giaa128_Supplemental_File [file giaa128_supplemental_file.zip › Supplementary_Figure1.png]
