# Supplementary material for: Trajectories, bifurcations, and pseudo-time in large clinical datasets: applications to myocardial infarction and diabetes data
Source: Gigascience. 2020 Nov 25;9(11):giaa128. doi: 10.1093/gigascience/giaa128 (PMC7688475; doi:10.1093/gigascience/giaa128)
Supplement: giaa128_Response_to_Reviewer_Comments_Original_Submission [file giaa128_response_to_reviewer_comments_original_submission.pdf]

Author's Response To Reviewer Comments

Close

Dear Editor,

We thank the reviewers of our manuscript for careful consideration and providing important suggestions and remarks. We think we addressed all of them in the revised version of the manuscript. Below you can find a detailed point-by-point answer to the reviewer's questions. The modifications introduced into the manuscript are highlighted in red.

In response to the editorial request: 'please register any new software application in the bio.tools and SciCrunch.org databases to receive RRID (Research Resource Identification Initiative ID) and biotoolsID identifiers.', we made a registration of the ClinTrajan package at SciCrunch.org, with the following RRID identifier: RRID:SCR_019018 and at bio.tools repository under the page https://bio.tools/clintrajan. Now we indicate this fact in the revised manuscript.

Best regards,
Andrei Zinovyev on behalf of the authors

Reviewer 1:

Remark: What is not clear from the manuscript is how these pseudo-time projections relate to real-time clinical trajectories. For example, the authors showcase the utility of this methodology in the context of myocardial infarction complications by using pseudo-time to define the risks of multiple different outcomes, including four distinct lethal outcomes, namely: progress of congestive heart failure; myocardial rupture; cardiogenic shock; and pulmonary edema. However, it is not clear from the manuscript whether it is possible to deliver a prognosis on, for example, 5-year survival for complications of myocardial infarction by using the pseudo-time plots shown in the manuscript? In addition, does geodesic distance from a branch point predict the severity of a particular disease complication? In this respect, I do note that lethality risk estimates are shown in the principal trees in Figure 2, and that lethality does correlate with cardiogenic shock and myocardial rupture, but the correlation with congestive heart failure and pulmonary oedema is not obvious from this figure. I wish to establish how the principal trees should be interpreted in a clinical environment, and consequently, I would like for the authors to detail the prognostic value of geodesic distance from branch point for each of the classes shown in Figure 2.

Answer:

We thank the Reviewer for pointing out to the missing aspect of using the extracted clinical trajectories for actual prediction of important clinical traits. We also totally agree that the method was missing more developed statistical tools for quantifying the risks of important clinical outcomes (such as lethality). In the case of myocardial infarction complication dataset, we can indeed try to predict the survival of a patient in the future, based on its current state. In the current version of the manuscript, we suggested adapting the standard survival analysis methodology for this purpose. Using this analysis, we computed the risks of lethal outcomes along distinct clinical trajectories, recapitulated in the form of estimated hazard functions of pseudotime. Moreover, we differentiated the contribution to the total risk of lethality from different types of complications. In addition, we used the survival regression analysis in order to identify lethality risk factors, which can be different along different clinical trajectories. As an example, we identified pre-existence of bronchial asthma in anamnesis as a risk factor for such infarction complications as congestive heart failure and asystole.

We introduced a new subsection 'Predicting survival and lethal risk factors' in the 'Results' part of the manuscript and 'Pseudotime survival analysis' subsection in the 'Methods' part and a new Figure 6, in which we illustrated the results of the survival-related analyses.

An important limitation of the suggested approach is having no possibility to connect the inferred pseudotime with physical time. In the survival prognosis we are using pseudotime, therefore, it is difficult to really give a 5-years survival prognosis, as the reviewer asked for. In the current version of the manuscript, we more carefully discuss this aspect and suggest what kind of information is needed to be collected in order to connect the pseudotime and physical time. In particular, in Discussion we now have the following paragraph:

Second approach to introduce physical time in the picture is using partially diachronic data as an additional annotation of a clinical dataset (the case of complete diachronic clinical data, representing longitudinal observations, is usually treated using a different and established set of approaches). One source of information which can be relatively easily obtained is identifying pairs of data points corresponding to two subsequent states of the same patient, and recording the time lapse between two states. For example, part of the patients in a clinical dataset can be returning to hospital, with a previous record included in the dataset, so this information must be available. If the number of such pairs is sufficiently large then one can try to learn a monotonic function Fk of pseudotime along each trajectory k, predicting the actual temporal label for each observation. Note that the connection between pseudotime and physical time can be different along different clinical trajectories. Moreover, the paired patient observation data can be used in the process of principal tree learning, by minimizing the number of paired patient observations belonging to distinct clinical trajectories.

Remark: In addition, in the study of diabetes, the authors report that it was possible to deliver pseudo-time plots from a publicly available dataset of 101766 records from 130 US hospitals. There are inherent issues with multi-site analysis as each hospital may have a slightly different means of capturing clinical data, and delivering accurate prognoses from such a diverse dataset is a challenge. Consequently, I see great value in the clinical trajectories of the large-scale diabetes dataset that are shown in Figure 6. However, I would like to know how the pseudotemporal dynamics of clinical variables shown in Figure 6C relate to more familiar diagnostic criteria, such as the level of hyperlipidemia and/or hypertension. Once again, I wish to establish the predictive value of the principal tree methodolody, and therefore I invite the authors to list the clinical correlates that a clinician should be able to predict by using geodesic distance from a branch point. In the specific case study of diabetes, I wish for the authors to comment on whether the pseudo-time approach outlined in the manuscript would have any predictive value in terms of blood pressure and/or or LDL cholesterol level.

Answer:

1) We totally agree with the reviewer that it would be nice to have more clinically relevant variables associated with pseudotemporal clinical trajectories in diabetes. We would be happy to add more relevant diagnostic criteria and clinical variables to analyse the diabetes trajectories, but we have to admit that this diabetes dataset does not contain such information (blood pressure and/or or LDL cholesterol level, information on hyperlipidemia and/or hypertension). Citing the above-mentioned reference: 'analyzing external data is more challenging than analysis of results of a carefully designed experiment or trial, because one has no impact on how and what type of information was collected. Nonetheless, it is important to utilize these huge amounts of data to find new information/knowledge that is possibly not available anywhere'. Only some diagnostic surrogates are available - such as the frequency of hospital admissions and prescribed drugs, from which it is quite difficult to infer the precise diagnostic data. One of the reasons for this might be indeed the multi-centric nature of the study such that only a common set of clinical features have been included in the merged dataset.

We would like to underline that our objective was to illustrate the work of the principal tree methodology using an example of large and publicly available clinical dataset, such that the analysis could be easily reproduced, which is the main requirement of GigaScience journal. Unfortunately, we found out that the number of large (having few thousands cases) and rich clinical datasets available in the public domain is still quite limited, with the diabetes and myocardial infarction datasets analysed in this paper being notable exceptions. This can be explained by the concerns of the authors of these datasets related to the issues of confidentiality and identifiability of clinical information. We see many interesting analyses being made on the clinical datasets which are not released in the public domain, and we think that the approach suggested by us will be useful for producing clinically relevant predictions - but given the closeness of the data, it will be difficult to reproduce.

Remark: In addition, as the impact of HbA1c measurement on Hospital Readmission Rates has already

been established (Strack et al. BioMed Res Int 2014:781670), can the authors explain the added value of generating principal trees to merely confirm this finding?

We thank the reviewer for pointing out the missing aspect of showing the added value of the principal trees with respect to the already known connection between the measurements of glycated hemoglobin and the early readmission rates. We looked closely at the relation between these two clinical features in distinct clinical trajectories, and found out that the global regression-based analysis performed in (Strack et al. BioMed Res Int 2014:781670) was hiding important heterogeneity in the dataset which can be revealed using the principal tree approach. In particular, we concluded that certain clinical trajectories can benefit from measuring HbA1c while in other clinical scenarios these measurements are less informative for anticipating the readmission rate.

In the revised version of the manuscript, we introduced a new subsection 'Trajectory-based analysis of the relation between early readmission and the measured glycated hemoglobin HbA1c' in the 'Results' part of the manuscript and a new Figure 8 reporting the results of this analysis.

Remark: Furthermore, I would also like the authors to comment on whether this approach has added value for multi-site clinical datasets.

Answer: We thank the reviewer for pointing out to the fact that many large clinical datasets can suffer from heterogeneity resulting from the multi-site nature of studies. Our approach, consisting in strong dimensionality reduction, indeed can smooth such heterogeneity up to certain extent and under some assumptions. In the revised version of the manuscript we introduced the following statement in the 'Possible implications' section:

The existing large clinical datasets are frequently collected as a result of multi-site studies. In case of strong artefacts and biases caused by application of significantly different practices for data collection or other factors, specific methods of correction should be applied, integrated into the data analysis or even in the study design\cite{Chen2012Design}. However, dimensionality reduction methods based on averaging can in principle partially compensate for data heterogeneity if it can be modeled as a mixture of independent site effects that remain relatively small compared to the ranges of variable variations along the clinical trajectories. ElPiGraph in this respect has advantages over much more rigid Principal Component Analysis, being a nonlinear generalization of it for the case of datasets with complex geometries\cite{Albergante2020}. However, this aspect of ElPiGraph requires a specific investigation.

Remark: Minor comments
The figure legends do not detail all the abbreviations used in the figures. The authors should list all abbreviations used in the manuscript.

Answer: All variable names and abbreviations used in the manuscript are now collected in the Dataset Description section. In particular, we introduced a new Table 1, where all variables shown in other Figures or mentioned in manuscript, are listed with explanations and grouped into the categories (e.g., a group of complication variables).

Reviewer 2:

Remark: 1. Not only the number of samples in the observational dataset but also the sample distributions make a great impact on whether accurate ElPiGraphs can be created. The authors may clarify this point.

Answer: We thank the reviewer for this remark. Indeed, the application of ElPiGraph is the most relevant for certain types of data point clouds, and less relevant for others, irrespectively of the total number of data points. In order to clarify this, we added the following paragraph in the Discussion:

Use of ElPiGraph is the most relevant in the case when the hypothetical probability density function underlying the multi-dimensional data is characterized by certain archetypal features. Recall that classical phenotyping is, in its essence, cluster analysis of data. Application of standard clustering methods assumes existence of lumps and peaks in the density function: therefore, clustering looks for a set of principal points \cite{Xu2008,Tarpey1996}.

Dynamical phenotyping has a different basic assumption that the point density is characterized by the existence of continuous one-dimensional ``ridges" which can diverge from or converge to each other in the data space. They can also connect local density peaks. In this case, the appropriate data approximation methods (such as ElPiGraph) look for principal curves and, more generally, branching principal trajectories, along which the data points are condensed\cite{Hastie1989, Albergante2020}. The relevance of such a data model for dynamical phenotyping follows from the nature of a complex dynamical process, underlying disease progression, which develops in physical time and is sampled in the space of clinical characteristics.

Remark: 2. The exact number of features in the myocardial infarction database is unclear.
In Page 3, it says ``110 features characterizing the clinical phenotypes'';
While in Page 4, it says ``the initial data space was formed by 123 variables'', and ``7 variables have been removed''; In Page 4, it also lists the features including ``84 binary, 9 continuous numerical, 22 ordinal, and 1 categorical'' which is 116 in total.

Answer: We thank the reviewer for attention to such details. There was indeed a misprint in the initial description of the dataset which contains 111 features characterizing the clinical phenotypes. In Page 3 it is now said 'It contains information about 1700 patients characterized by 111 features describing the clinical phenotypes and 12 features representing possible complications of the myocardial infarction disease (123 features in total).' After removal of 7 having too many missing values, we are left with 116 variables classified into different types in Page 4.

Remark: 3. In the first part of the Analyses Section, an extra period exists in the list, after ``associating the segments to clinical variables''.

Answer: This has been corrected

Remark: 4. For the analyses on myocardial infarction study, it may be better to reorder the paragraphs as 1) Quantification of nominal values ..., 2) Constructing ElPiTree, 3) Assigning data point classes, followed by the rest.

Answer: We are thankful for this proposition, which makes sense to us. The sections have been reordered.

Remark: 5. In Page 11, ``disandvatage'' should be ``disadvantage''; ``Then the standard SVD or order k'' should be ``of order k''.

Answer: This has been corrected

Reviewer 3:

Remark: The only real comment that I have is showing a real world example of how this works, this could be added to an online supplement.

Answer: In the revised version of the manuscript we added a step-by-step tutorial based on the analysis of the myocardial infarction complications dataset, which is now provided at https://github.com/auranic/ClinTrajan

Close
