# Supplementary material for: Trajectories, bifurcations, and pseudo-time in large clinical datasets: applications to myocardial infarction and diabetes data
Source: Gigascience. 2020 Nov 25;9(11):giaa128. doi: 10.1093/gigascience/giaa128 (PMC7688475; doi:10.1093/gigascience/giaa128)
Supplement: giaa128_Response_to_Reviewer_Comments_Revision_1 [file giaa128_response_to_reviewer_comments_revision_1.pdf]

Author's Response To Reviewer Comments

Close

Dear Editor,

We addressed the issues indicated as comments in the edited pdf file (list of abbreviations and adding an extra reference to the GigaDB entry for this manuscript)

Best regards,
Andrei Zinovyev

Close
