# Supplementary material for: Trajectories, bifurcations, and pseudo-time in large clinical datasets: applications to myocardial infarction and diabetes data
Source: Gigascience. 2020 Nov 25;9(11):giaa128. doi: 10.1093/gigascience/giaa128 (PMC7688475; doi:10.1093/gigascience/giaa128)
Supplement: giaa128_Reviewer_1_Report_Original_Submission — Chris Armit -- 7/15/2020 Reviewed [file giaa128_reviewer_1_report_original_submission.pdf]

Reviewer Report

Title: Trajectories, bifurcations and pseudotime in large clinical datasets: applications to myocardial infarction and diabetes data

Version: Original Submission **Date:** 7/15/2020

Reviewer name: Chris Armit

Reviewer Comments to Author:

This novel Research Article utilises elastic principal trees (EPT) - a non-linear generalisation of Principal Component Analysis (PCA) - as a means of generating clinical trajectories of complications of myocardial infarction and diabetes. The authors define clinical trajectories as "a clinically relevant sequence of ordered patient phenotypes representing consecutive states of a developing disease and leading to some final state" and they utilise geodesic distance, which they refer to as "pseudo-time" quantification as a means of predicting disease outcome. Whereas there have been recent gene expression studies in single cells and bulk tissue that utilise the concept of pseudo-time as a means of assessing the relative progression of individuals along a trajectory of interest such as disease progression (Campbell & Yau, Nature Communications 2018;9,2442 (2018); Saelens et al., Nature Biotechnology 2019;37(5):547-554), this is the first time I have encountered this concept applied to clinical data. The principal tree methodology utilised in this computational study is a set of principal curves assembled in a tree-like structure and characterised by branching topology, and by quantifying the geodesic distances the authors arrive at a measure of "pseudo-time".

Major comments

What is not clear from the manuscript is how these pseudo-time projections relate to real-time clinical trajectories. For example, the authors showcase the utility of this methodology in the context of myocardial infarction complications by using pseudo-time to define the risks of multiple different outcomes, including four distinct lethal outcomes, namely: progress of congestive heart failure; myocardial rupture; cardiogenic shock; and pulmonary edema. However, it is not clear from the manuscript whether it is possible to deliver a prognosis on, for example, 5-year survival for complications of myocardial infarction by using the pseudo-time plots shown in the manuscript? In addition, does geodesic distance from a branch point predict the severity of a particular disease complication? In this respect, I do note that lethality risk estimates are shown in the principal trees in Figure 2, and that lethality does correlate with cardiogenic shock and myocardial rupture, but the correlation with congestive heart failure and pulmonary oedema is not obvious from this figure. I wish to establish how the principal trees should be interpreted in a clinical environment, and consequently, I would like for the authors to detail the prognostic value of geodesic distance from branch point for each of the classes shown in Figure 2.

In addition, in the study of diabetes, the authors report that it was possible to deliver pseudo-time plots from a publicly available dataset of 101766 records from 130 US hospitals. There are inherent issues with multi-site analysis as each hospital may have a slightly different means of capturing clinical data, and delivering accurate prognoses from such a diverse dataset is a challenge. Consequently, I see great

value in the clinical trajectories of the large-scale diabetes dataset that are shown in Figure 6. However, I would like to know how the pseudotemporal dynamics of clinical variables shown in Figure 6C relate to more familiar diagnostic criteria, such as the level of hyperlipidemia and/or hypertension. Once again, I wish to establish the predictive value of the principal tree methodolody, and therefore I invite the authors to list the clinical correlates that a clinician should be able to predict by using geodesic distance from a branch point. In the specific case study of diabetes, I wish for the authors to comment on whether the pseudo-time approach outlined in the manuscript would have any predictive value in terms of blood pressure and/or or LDL cholesterol level. In addition, as the impact of HbA1c measurement on Hospital Readmission Rates has already been established (Strack et al. BioMed Res Int 2014:781670), can the authors explain the added value of generating principal trees to merely confirm this finding? Furthermore, I would also like the authors to comment on whether this approach has added value for multi-site clinical datasets.
Minor comments
The figure legends do not detail all the abbreviations used in the figures. The authors should list all abbreviations used in the manuscript.

Methods

Are the methods appropriate to the aims of the study, are they well described, and are necessary controls included? Choose an item.

Conclusions

Are the conclusions adequately supported by the data shown? Choose an item.

Reporting Standards

Does the manuscript adhere to the journal's guidelines on minimum standards of reporting? Choose an item.

Choose an item.

Statistics

Are you able to assess all statistics in the manuscript, including the appropriateness of statistical tests used? Choose an item.

Quality of Written English

Please indicate the quality of language in the manuscript: Choose an item.

Declaration of Competing Interests

Please complete a declaration of competing interests, considering the following questions:

- Have you in the past five years received reimbursements, fees, funding, or salary from an organisation that may in any way gain or lose financially from the publication of this manuscript, either now or in the future?
- Do you hold any stocks or shares in an organisation that may in any way gain or lose financially from the publication of this manuscript, either now or in the future?
- Do you hold or are you currently applying for any patents relating to the content of the manuscript?
- Have you received reimbursements, fees, funding, or salary from an organization that holds or has applied for patents relating to the content of the manuscript?
- Do you have any other financial competing interests?
- Do you have any non-financial competing interests in relation to this paper?

If you can answer no to all of the above, write 'I declare that I have no competing interests' below. If your reply is yes to any, please give details below.

I declare that I have no competing interests.

I agree to the open peer review policy of the journal. I understand that my name will be included on my report to the authors and, if the manuscript is accepted for publication, my named report including any attachments I upload will be posted on the website along with the authors' responses. I agree for my report to be made available under an Open Access Creative Commons CC-BY license (http://creativecommons.org/licenses/by/4.0/). I understand that any comments which I do not wish to be included in my named report can be included as confidential comments to the editors, which will not be published.

Choose an item.

To further support our reviewers, we have joined with Publons, where you can gain additional credit to further highlight your hard work (see: https://publons.com/journal/530/gigascience). On publication of this paper, your review will be automatically added to Publons, you can then choose whether or not to claim your Publons credit. I understand this statement.

Yes Choose an item.
