# Supplementary material for: Trajectories, bifurcations, and pseudo-time in large clinical datasets: applications to myocardial infarction and diabetes data
Source: Gigascience. 2020 Nov 25;9(11):giaa128. doi: 10.1093/gigascience/giaa128 (PMC7688475; doi:10.1093/gigascience/giaa128)
Supplement: giaa128_Reviewer_2_Report_Original_Submission — Zhengping Che -- 8/5/2020 Reviewed [file giaa128_reviewer_2_report_original_submission.pdf]

Reviewer Report

Title: Trajectories, bifurcations and pseudotime in large clinical datasets: applications to myocardial infarction and diabetes data

Version: Original Submission **Date:** 8/5/2020

Reviewer name: Zhengping Che

Reviewer Comments to Author:

This manuscript proposes a method for analyzing disease progression based on modeling the clinical trajectory and quantizing the pseudotime of the patient. The proposed method is based on creating the elastic principal graphs (ElPiGraphs) and is with an implementation in Python package ClinTrajan. Two public observational clinical datasets on myocardial infarction with complications and diabetes readmission are analyzed, and the proposed method has been well demonstrated.
This paper is generally well written, and each step of its methodology has been motivated and described moderately. The result visualizations, along with analyses, are impressive and convincing. Some detailed comments are listed below.
1. Not only the number of samples in the observational dataset but also the sample distributions make a great impact on whether accurate ElPiGraphs can be created. The authors may clarify this point.
2. The exact number of features in the myocardial infarction database is unclear.
In Page 3, it says ``110 features characterizing the clinical phenotypes'';
While in Page 4, it says ``the initial data space was formed by 123 variables'', and ``7 variables have been removed''; In Page 4, it also lists the features including ``84 binary, 9 continuous numerical, 22 ordinal, and 1 categorical'' which is 116 in total.
3. In the first part of the Analyses Section, an extra period exists in the list, after ``associating the segments to clinical variables''.
4. For the analyses on myocardial infarction study, it may be better to reorder the paragraphs as 1) Quantification of nominal values ..., 2) Constructing ElPiTree, 3) Assigning data point classes, followed by the rest.
5. In Page 11, ``disandvatage'' should be ``disadvantage''; ``Then the standard SVD or order k'' should be ``of order k''.

Methods

Are the methods appropriate to the aims of the study, are they well described, and are necessary controls included? Choose an item.

Conclusions

Are the conclusions adequately supported by the data shown? Choose an item.

Reporting Standards

Does the manuscript adhere to the journal's guidelines on [minimum standards of reporting?]() Choose an item.

Choose an item.

Statistics

Are you able to assess all statistics in the manuscript, including the appropriateness of statistical tests used? Choose an item.

Quality of Written English

Please indicate the quality of language in the manuscript: Choose an item.

Declaration of Competing Interests

Please complete a declaration of competing interests, considering the following questions:

- Have you in the past five years received reimbursements, fees, funding, or salary from an organisation that may in any way gain or lose financially from the publication of this manuscript, either now or in the future?
- Do you hold any stocks or shares in an organisation that may in any way gain or lose financially from the publication of this manuscript, either now or in the future?
- Do you hold or are you currently applying for any patents relating to the content of the manuscript?
- Have you received reimbursements, fees, funding, or salary from an organization that holds or has applied for patents relating to the content of the manuscript?
- Do you have any other financial competing interests?
- Do you have any non-financial competing interests in relation to this paper?

If you can answer no to all of the above, write 'I declare that I have no competing interests' below. If your reply is yes to any, please give details below.

I declare that I have no competing interests.

I agree to the open peer review policy of the journal. I understand that my name will be included on my report to the authors and, if the manuscript is accepted for publication, my named report including any attachments I upload will be posted on the website along with the authors' responses. I agree for my report to be made available under an Open Access Creative Commons CC-BY license (http://creativecommons.org/licenses/by/4.0/). I understand that any comments which I do not wish to be included in my named report can be included as confidential comments to the editors, which will not be published.

Choose an item.

To further support our reviewers, we have joined with Publons, where you can gain additional credit to further highlight your hard work (see: https://publons.com/journal/530/gigascience). On publication of this paper, your review will be automatically added to Publons, you can then choose whether or not to claim your Publons credit. I understand this statement.

Yes Choose an item.
